# Sexual Fate Change of XX Germ Cells Caused by the Deletion of SMAD4 and STRA8 Independent of Somatic Sex Reprogramming

**DOI:** 10.1371/journal.pbio.1002553

**Published:** 2016-09-08

**Authors:** Quan Wu, Kurumi Fukuda, Yuzuru Kato, Zhi Zhou, Chu-Xia Deng, Yumiko Saga

**Affiliations:** 1 Department of Genetics, Sokendai, Mishima, Japan; 2 Division of Mammalian Development, National Institute of Genetics, Mishima, Japan; 3 Faculty of Health Sciences, University of Macau, Macau SAR, China; 4 Department of Biological Sciences, Graduate School of Science, The University of Tokyo, Tokyo, Japan; Cornell University, UNITED STATES

## Abstract

The differential programming of sperm and eggs in gonads is a fundamental topic in reproductive biology. Although the sexual fate of germ cells is believed to be determined by signaling factors from sexually differentiated somatic cells in fetal gonads, the molecular mechanism that determines germ cell fate is poorly understood. Herein, we show that mothers against decapentaplegic homolog 4 (SMAD4) in germ cells is required for female-type differentiation. Germ cells in *Smad4*-deficient ovaries respond to retinoic acid signaling but fail to undergo meiotic prophase I, which coincides with the weaker expression of genes required for follicular formation, indicating that SMAD4 signaling is essential for oocyte differentiation and meiotic progression. Intriguingly, germline-specific deletion of *Smad4* in *Stra8*-null female germ cells resulted in the up-regulation of genes required for male gonocyte differentiation, including *Nanos2* and *PLZF*, suggesting the initiation of male-type differentiation in ovaries. Moreover, our transcriptome analyses of mutant ovaries revealed that the sex change phenotype is achieved without global gene expression changes in somatic cells. Our results demonstrate that SMAD4 and STRA8 are essential factors that regulate the female fate of germ cells.

## Abstract 2

Double ablation of SMAD4 and STRA8 causes female-to-male switching of XX germ cells without affecting somatic cell fate. This suggests that SMAD4 and STRA8 are essential intrinsic factors that determine the female fate of germ cells, collaborating to suppress expression of male genes.

## Author Summary

Mammalian sex depends on a male-specific gene, sex-determining region Y (SRY), which is located on the Y chromosome. Individuals lacking this gene will develop as female. Accordingly, germ cell fate also changes from male to female in the absence of SRY. Therefore, it is thought that somatic cells regulate germ cells to become sperm or oocytes. However, it is largely unknown what factor is responsible for sexual fate determination in germ cells. In fetal ovaries, retinoic acid (RA) initiates STRA8 expression in germ cells and induces meiosis. Female germ cells without STRA8 fail to enter meiosis but still progress to oogenesis and form oocyte-like cells, indicating that RA is not the regulator of oogenesis. Here, we found that female germ cells lacking both SMAD4 and STRA8 (but not a single knockout) develop as male gonocyte-like cells in ovaries, indicating that these two factors work as female germ cell determinants. To our surprise, the sexual fate switch observed in the double knockout ovary is not accompanied by gene expression changes in somatic cells, revealing the unexpected finding that somatic factors controlled by SRY are dispensable for the upregulation of male-specific genes in germ cells.

## Introduction

Primordial germ cells (PGCs) that emerge in the early mouse embryo have the capability to become either spermatocytes or oocytes and are enclosed by somatic cells in embryonic testes or ovaries. The sexual fate of germ cells in gonads is thought to be determined by factors derived from somatic cells because somatic sex determination precedes germ cell sex determination, and sex reversal in somatic cells leads to sex reversal in germ cells [1]. However, when and how germ cells receive such signals, and the determinants of germ cell sex, remain unknown. Understanding the mechanism of sex determination in germ cells requires elucidation of the pathways associated with male and female differentiation events.

The earliest sign of sexual differentiation in male germ cells is the entry into cell cycle arrest that is likely due to the suppression of retinoic acid (RA) signaling caused by upregulation of the RA-metabolizing enzyme CYP26B1 in somatic cells [2–4]. Simultaneously, the male-specific factor NANOS2 is induced by mothers against decapentaplegic homolog (Smad) 2 signaling in germ cells, and the expression is maintained during the embryonic stage [5–8]. The sex of germ cells in testes is determined by NANOS2 function, as the absence of NANOS2 impedes male differentiation and results in the induction of female characteristics, such as meiotic initiation and upregulation of genes associated with oocyte differentiation [9,10]. Inversely, if *Nanos2* is ectopically induced in female germ cells, the cells fail to enter meiosis and begin male-specific gene expression, such as DNA methyltransferase 3-like protein (*Dnmt3L*) and *MIWI2*, which are required for male-specific de novo DNA methylation in embryonic testes [10–13]. The somatic factor sex-determining region Y (SRY) and its downstream effectors SRY-box (SOX) 9 and fibroblast growth factor 9 (FGF9) are essential for the male fate decision because their deletions lead to male-to-female sex reversal, germ cells included [14–19]. Among these factors, FGF9 is considered to work directly in germ cells to induce *Nanos2* [16,20]. However, a recent report showed that the deletion of a female-specific gene, wingless-related MMTV integration site 4 (*WNT4*), in *Fgf9*-null testes rescues the switch from male to female, which suggests that FGF9 acts by suppressing WNT4 expression rather than by actively directing male fate [21]. Thus, the signal that induces the male fate decision remains unknown.

The sexual fate of germ cells in ovaries appears to be induced by two distinct processes—meiosis and oocyte differentiation—that proceed simultaneously [22]. Upon receiving an RA signal, XX germ cells express genes such as stimulated by retinoic acid 8 (*Stra8*) and *Rec8* [2,23,24]. After pre-meiotic DNA replication controlled by STRA8, germ cells enter meiotic prophase I, during which homologous chromosome pairing and recombination occurs in a series of stages: leptotene, zygotene, pachytene, and diplotene. Therefore, sexual differentiation in the ovary is associated with meiotic initiation, a process that is never observed in wild-type testes during the embryonic stage. In *Stra8*-deficient ovaries, XX germ cells enter neither meiosis nor mitosis and retain DNA content as 2N [23]. However, a recent study showed that oocyte-like cells are generated without premeiotic replication in *Stra8*-deficient ovaries, which indicates that the feminization of germ cells occurs independent of meiosis and that factors other than RA must be involved in the induction of oocyte differentiation [22]. FOXL2 and WNT4 are possible somatic factors required for female fate determination, as evidenced by the sex reversal of XX PGCs caused by the deletion of *Foxl2* and *Wnt4* [25]. However, it is controversial whether the effector of Wnt signaling, β-catenin, functions in somatic cells or germ cells [26–28]. Therefore, the somatic factors downstream of FOXL2 and WNT4 signals that directly induce oocyte differentiation are unclear.

To clarify the signals that lead to the sexual determination of germ cells, we have focused on the transforming growth factor β (TGFβ) signaling pathway. We have shown that nodal/activin signaling is activated in germ cells in embryonic testis but not in ovaries, and is required for the induction of the male determinant NANOS2 [5,8]. In this study, we investigated the possible role of bone morphogenetic protein (BMP) signaling in the sexual differentiation of germ cells in ovaries because a previous study suggested that *Bmp2* expression was under the control of Wnt signaling, although the function of BMP2 remains unknown [29]. BMP signaling is mediated by SMAD 1, 5, or 8 and their common mediator Co-SMAD4 [30]. To determine the function of BMP signaling, we deleted *Smad4* in a germ-line-specific manner. We here provide evidence that Smad4 signaling plays essential roles in the induction of oocyte-specific regulators as well as in meiotic progression. Notably, the suppression of Smad4 and RA signaling in germ cells is sufficient to direct the cells to take a male pathway even in the absence of male somatic cells.

## Results

### Smad4 Signaling Is Required for Germ Cell Survival in the Fetal Ovary

To investigate any involvement of BMP signaling in the fetal ovary, we used immunostaining to examine the expression pattern of phosphorylated SMAD (pSMAD) 1/5/8. pSMAD1/5/8 was detected in both germ and somatic cells in ovaries from E11.5 to E13.5, suggesting that BMP signaling is activated in both cell types (S1A Fig). pSMAD1/5/8 requires SMAD4 to activate downstream genes even though these factors can enter the nucleus upon ligand binding [31]. Thus, we used a floxed allele of *Smad4* encoding the Co-SMAD protein to analyze the function of BMP signaling in ovaries. Although SMAD4 may mediate other TGFβ-signaling pathways mediated by SMAD2 and 3, previous studies have shown that phosphorylated SMAD2 was not detectable in ovaries at this stage [5,32]. Therefore, we reasoned that the deletion of *Smad4* may disrupt pSMAD1/5/8-dependent BMP signaling rather than pSMAD2/3-dependent signaling in the ovary, but we do not exclude the possibility that SMAD4 is also involved in other signaling pathways.

**Fig 1:**
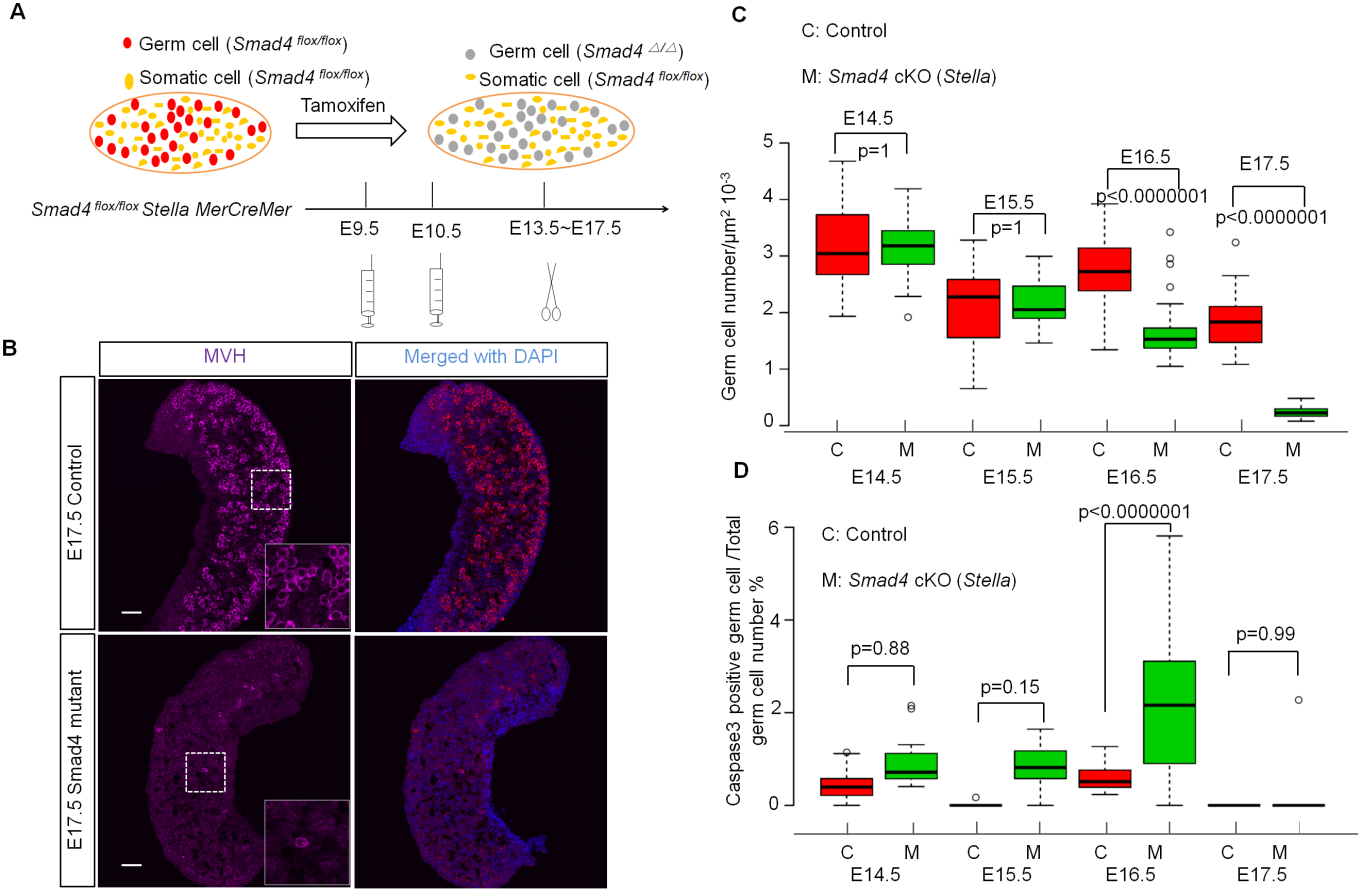
Smad4 signaling is required for germ cell survival. (A) Scheme of germ-cell-specific knockout strategy. Tamoxifen was injected at E9.5 and E10.5, and ovaries were recovered at indicated stages. (B) Representative image of E17.5 *Smad4 (Stella)* mutant and control ovarian tissue sections stained for MVH and DAPI. (C,D) Temporal changes of germ cell number (C) and cleaved-caspase3 positive germ cells (D) in control and *Smad4 (Stella)* mutant ovaries. Sample numbers used for counting were indicated in S1 Data. Significance was assessed using one-way ANOVA followed by Tukey’s post-hoc tests for selected pairs of genotypes. Scale bars: 50 μm. We used *Smad*^*flox/flox*^, *Smad4*^*flox/+*^, and *Smad4*^*flox/+*^ *StellaMerCreMer* ovaries as the control.

*Smad4* was deleted in a germ cell-specific manner by using a transgenic *Stella-MerCreMer* line [33], in which Cre recombinase activity was induced by tamoxifen (TM) administration under the regulation of the element that controls *Stella* (a gene specifically expressed in germ cells) expression. We injected TM at E9.5 and E10.5 to delete *Smad4* in germ cells (Fig 1A). We refer to this mouse model as the *Smad4 (Stella)* mutant unless otherwise specified. The efficiency of Cre activity was variable, but was as high as 80% based on the reporter gene expression (S1B Fig). We found that the germ cell number decreased in the mutant ovaries at E16.5 and E17.5 (Fig 1B and 1C). As the reduction of female germ cells was correlated with the increase of cleaved-caspase3 positive germ cells at E16.5 (Fig 1D), it is likely that the observed germ cell loss was due to apoptotic cell death. These results indicate that *Smad4* is required for female germ cell survival.

### Crucial Role of Smad4 Signaling in Female Germ Cell Differentiation Independent of RA Signaling

**Fig 2:**
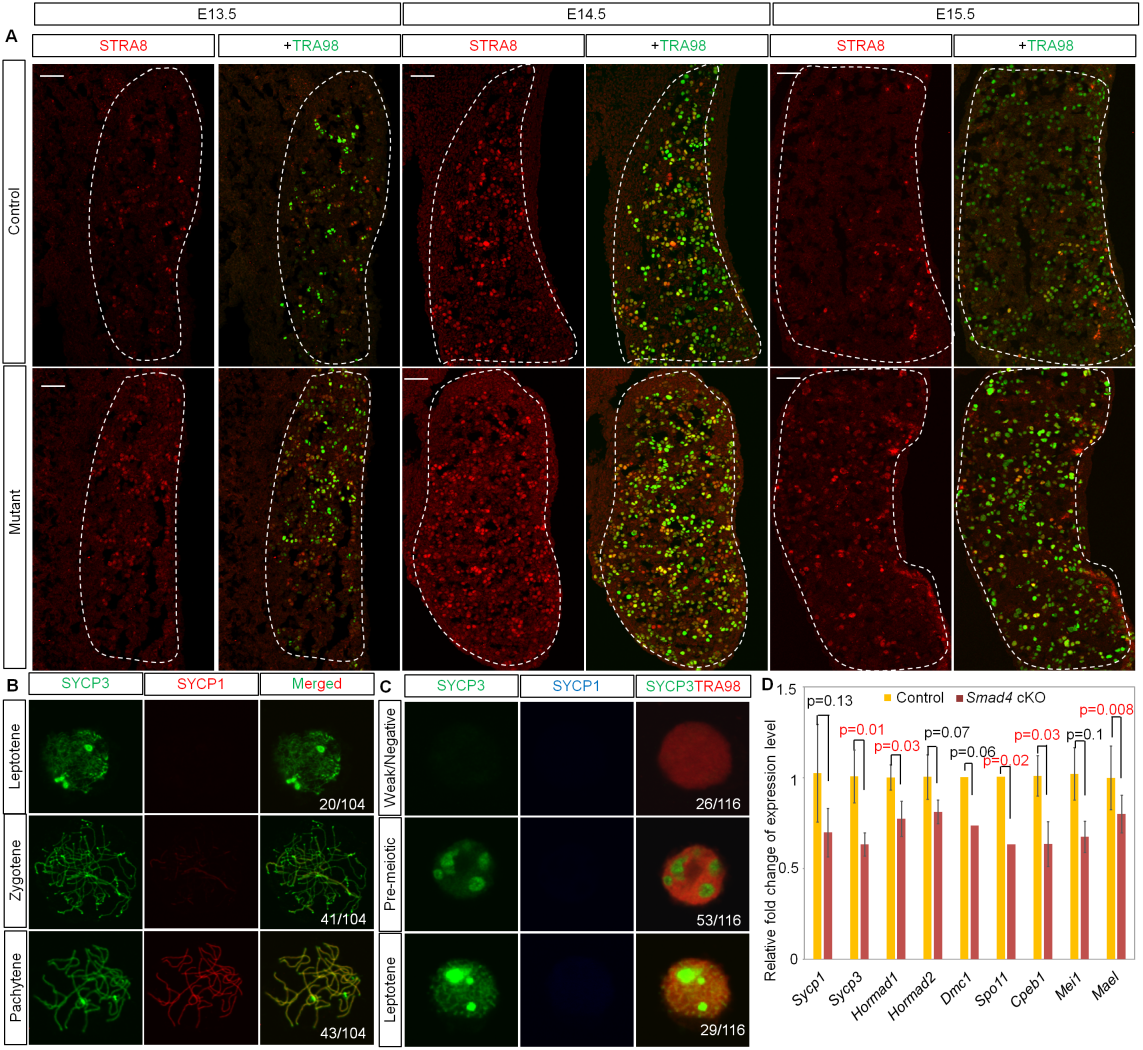
Initiation of meiosis but failure of meiotic progression in *Smad4 (Stella)* germ cells. (A) Representative image of the indicated ovary sections stained for STRA8 and TRA98 at E13.5, E14.5, and E15.5 in control and *Smad4 (Stella)* mutant ovaries. Ovaries were marked by dotted lines. Scale bars: 50 μm. (B) Representative images of leptotene, zygotene, and pachytene stage oocytes stained with SYCP3 and SYCP1 in control ovaries at E15.5. Number of oocytes in each stage is shown. (C) Representative images of different patterns of germ cells stained with SYCP3 and SYCP1 in *Smad4 (Stella)* ovaries at E15.5. Number of germ cells in each pattern is shown. (D) Expression level of meiosis-related genes in control and *Smad4 (Stella)* ovaries at E14.5 (*n* = 3 each). Data was extracted from microarray analysis (see S1 Data). Data is shown by a bar graph with *p*-value calculated by Student’s *t* test. Error bars indicate standard deviation.

To investigate the cause of germ cell death and understand the function of SMAD4, we examined the gene expression changes that occurred before apoptosis. As female differentiation consists of meiosis and oocyte differentiation pathways, we first examined the expression of *Stra8*, a gene involved in pre-meiotic DNA replication [3,23]. In normal ovaries, STRA8 is expressed in germ cells from E13.5, and most of the germ cells lost this expression at E15.5 due to the progression of meiotic prophase I (Fig 2A). Even in *Smad4 (Stella)* mutant ovaries, STRA8 expression was observed from E13.5 as well, indicating that the deletion of *Smad4* does not interfere with RA signaling. However, its expression was retained up to E15.5 (Fig 2A), suggesting that *Smad4*-deficiency influences the progression of meiosis.

To evaluate *Smad4*-deficiency defects after the initiation of *Stra8* expression, we performed a cytological examination with *Smad4 (Stella)* mice at E15.5. In control ovaries, immunodetection of synaptonemal complex proteins (SYCPs) 1 and 3 in meiocyte spreads revealed normal meiotic progression: germ cells were found at leptotene (20/104 = 19.2%), zygotene (41/104 = 39.4%), or pachytene (43/104 = 41.3%) stages (Fig 2B). However, in *Smad4 (Stella)* ovaries, we found that 22.4% (26/116) of TRA98 (a germ cell marker)-positive cells showed weak or negative SYCP3 signals (Fig 2C, upper panel). Furthermore, SYCP3 localized in nucleoli in 45.7% (53/116) of *Smad4(Stella)* germ cells (Fig 2C, middle panel), similar to that observed in pre-meiotic female germ cells and *Stra8*-null germ cells at E15.5 [23]. The remaining 25.0% (29/116) and 6.9% (8/116) of *Smad4 (Stella)* germ cells were at the leptotene stage lacking the central synaptonemal complex (SC) marker SYCP1 (Fig 2C, lower panel) or at the zygotene stage, respectively. No pachytene-stage germ cells were observed in *Smad4*-deficient ovaries. The failure of meiosis progression was also examined by expression analyses of meiosis-related genes at E14.5. These genes included *Sycp1*, *Sycp3*, *Cpeb1*, *Mei1*, *Hormad1*, and *Hormad2* [34–37], which are related to the formation of SC protein and *Spo11*, *Dmc1*, and *Mael*, which are required for the induction, formation, and repair of double strand breaks and repression of transposable elements [38–40]. The expression levels of five out of nine genes were lower in the mutant than in the controls (Fig 2D). These results suggest that Smad4 signaling is required for meiotic progression independent of RA signaling.

**Fig 3:**
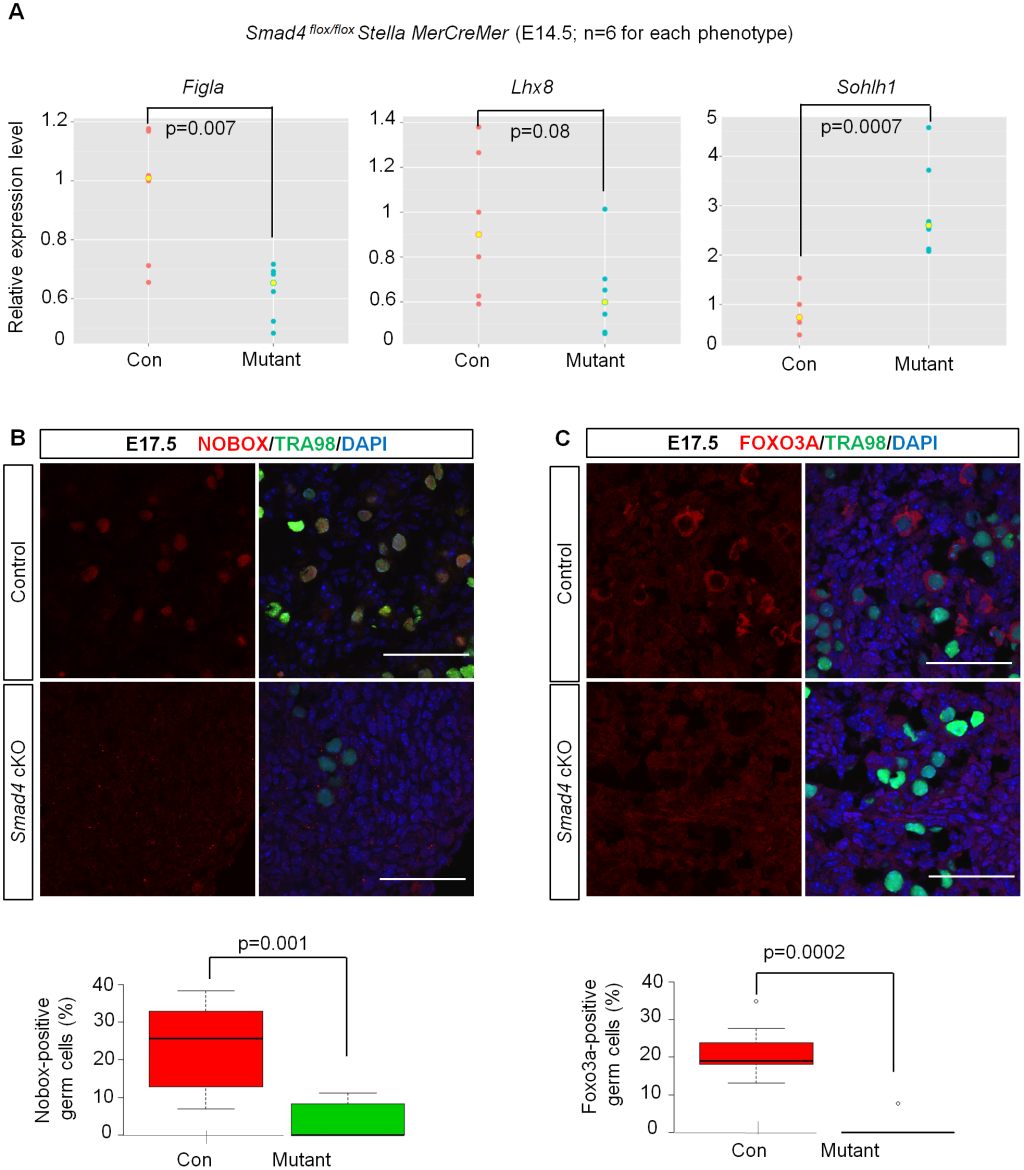
Smad4 signaling is required for the expression of genes involved in follicular formation. (A) Expression levels of the indicated genes were compared by RT-qPCR in E14.5 control and *Smad4 (Stella)* ovaries (*n* = 6), and the expression levels of the indicated genes were normalized to that of *mouse vasa homolog* (*Mvh*). Expression level of genes in one of the control ovaries was set as 1. Significance was assessed by the Student’s *t* test. (B,C) Representative images of E17.5 *Smad4 (Stella)* and control ovarian tissue sections stained for NOBOX and TRA98 (B), and FOXO3A and TRA98 (C) are shown. The quantitative data are shown below (*n* = 3). Significance was assessed by the Student’s *t* test. Underlying data is available in S1 Data. Scale bars: 50 μm.

Next, we investigated the expression of genes involved in oocyte differentiation. We found that the expression of *Figla*, a key transcription factor required for primordial follicle formation, was decreased in *Smad4 (Stella)*-mutant ovaries; however, two other regulators, *Lhx8* and *Sohlh1* [41], were either not decreased (*Lhx8*) or significantly increased (*Sohlh1*) at E14.5 (Fig 3A). Although apoptosis occurred in *Smad4*-null germ cells at E16.5, some germ cells still survived. However, the number of newborn ovary homeobox protein (NOBOX)- and Forkhead box O3a (FOXO3A)-positive germ cells was fewer in *Smad4*-deficient ovaries than in the control at E17.5 (Fig 3B and 3C). These data suggest that SMAD4 plays a role in oogenic gene expression, although it is unclear whether SMAD4 directly controls these gene expressions or not.

### SMAD4 in Somatic Cells Does Not Contribute to Female-Type Differentiation of Germ Cells

Next, we examined whether SMAD4 in somatic cells contributes to the female differentiation of germ cells. To delete *Smad4* from somatic cells, we used *WT1-CreERT2* mice. In this mouse line, *CreERT2* was inserted into the *Wt1* locus, and WT1 is expressed in genital ridges beginning at around E9.5 [42,43]. We injected TM into *Smad4*^*flox/flox*^/*WT1-CreERT2* mice at E10.5 and E11.5 to delete *Smad4* in somatic cells. *Smad4* expression was repressed, but this had no effect on *Figla* and *Lhx8* expression; however, *Sohlh1* expression was slightly upregulated at E14.5 (S2A and S2B Fig). As most embryos died at E15.5 under our experimental conditions, we therefore cultured embryonic gonads for 3 days from E14.5 and examined the germ cell characteristics. We found that the differentiation of female germ cells was unaffected based on a comparison of NOBOX-positive germ cells in control and mutant ovaries (S2C Fig). In contrast, ubiquitous deletion of *Smad4* in *Smad4*^*flox/flox*^/*Rosa-CreERT2* ovaries showed results similar to those observed in *Smad4 (Stella)* ovaries (S3 Fig). Expression of many meiotic marker genes as well as genes involved in follicle formation (*Lhx8* and *Figla*) was downregulated, while *Stra8* and *Rec8* expressions were not decreased (S3B Fig). Therefore, we concluded that Smad4 signaling acts in germ cells rather than somatic cells to control differentiation of germ cells in ovaries.

### Suppression of Smad4 and RA Signaling Induces Ectopic NANOS2 Expression in the Ovary

**Fig 4:**
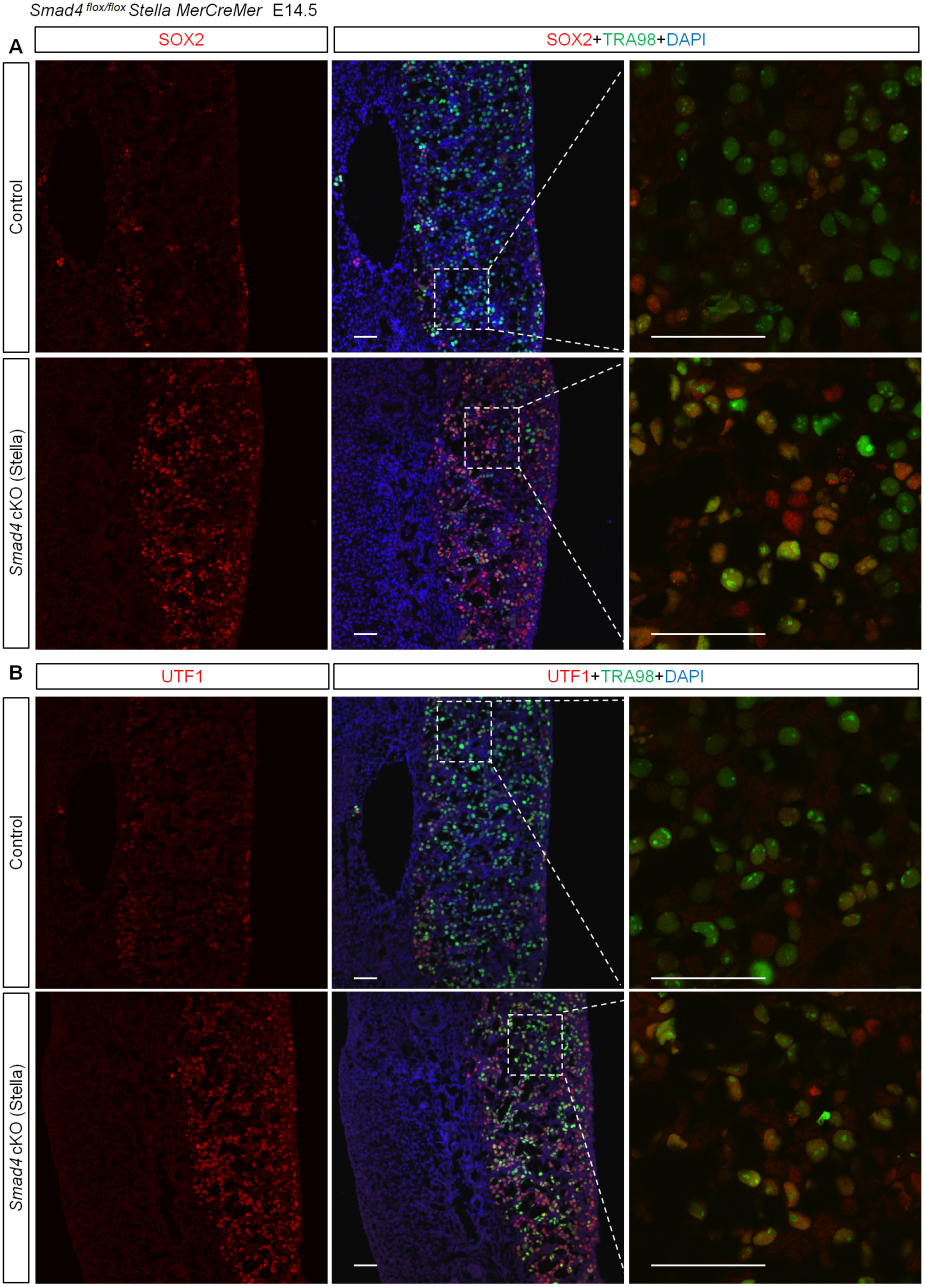
Maintenance of pluripotency gene expression in XX germ cells in the absence of SMAD4. (A,B) Immunohistochemical detection of SOX2/TRA98 (A) and UTF1/TRA98 (B) in the E14.5 control and *Smad4 (Stella)* ovaries. Scale bars: 50 μm.

To further examine the function of SMAD4 in the early germ cell differentiation pathway, we examined pluripotency-related genes. In germ cells of normal ovaries, the expression of the pluripotency-related genes *Sox2*, octamer-binding transcription factor 4 (*Oct4*), *Nanog*, and undifferentiated embryonic cell transcription factor 1 (UTF1) is downregulated at E14.5 accompanied by the upregulation of genes involved in female differentiation, which indicates that the downregulation is associated with female differentiation (Fig 4) [44,45]. In *Smad4*-deficient germ cells, however, these gene expressions were retained at high levels (Fig 4 and S3D Fig), indicating that SMAD4 is involved in the suppression of pluripotency-related genes upon entering a female differentiation pathway.

**Fig 5:**
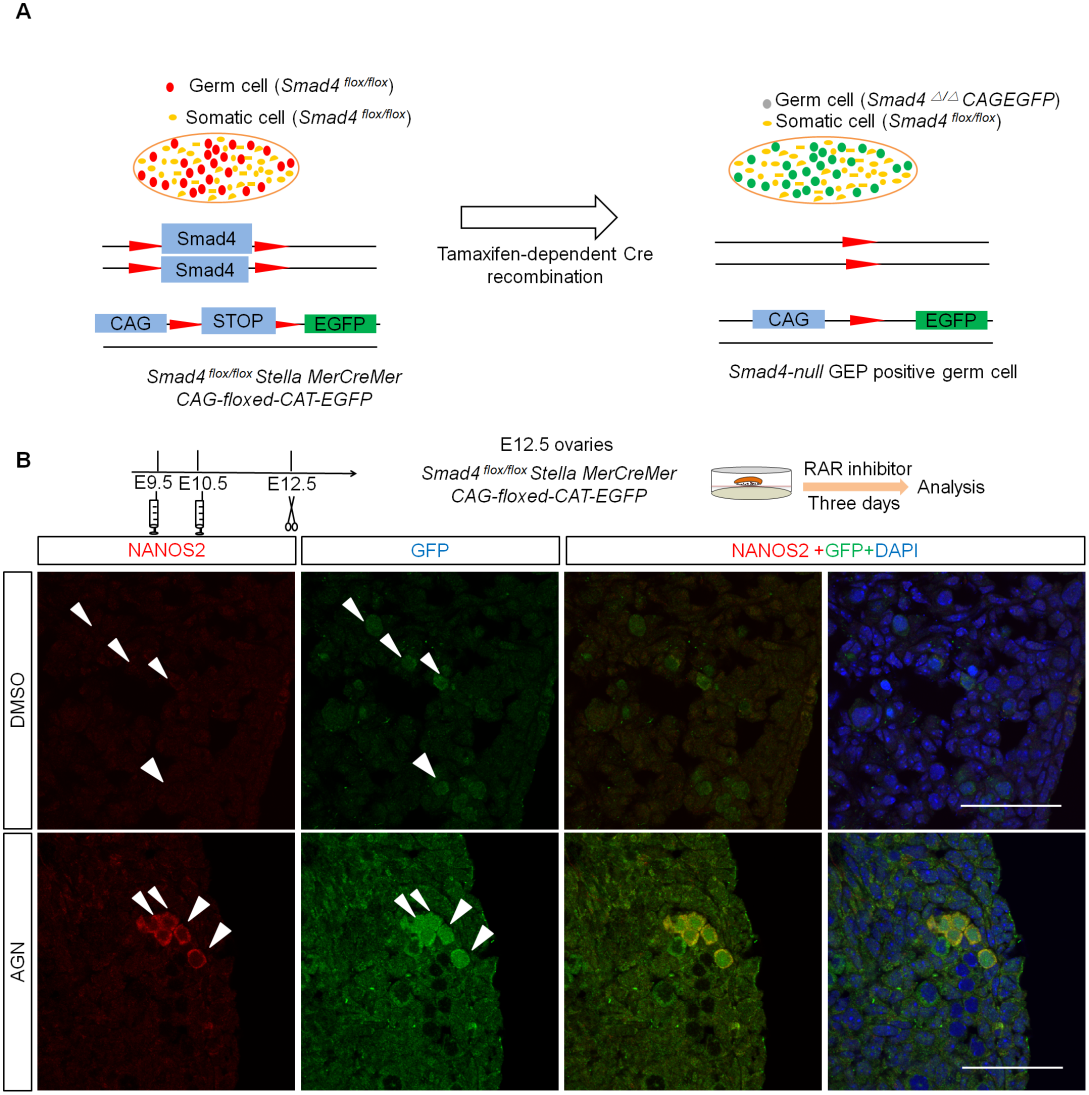
NANOS2 upregulation occurred in *Smad4*-null germ cells in the absence of RA signaling. (A) Scheme of strategy to trace the fate of *Smad4-null* germ cells. Deletion of *Smad4* by Cre recombination induces the expression of GFP, indicating *Smad4*-null germ cells. (B) Representative image of sections from *Smad4 (Stella)* ovaries after DMSO or AGN193109 treatment stained for NANOS2 and GFP. White arrows indicate GFP-positive cells. Ovaries were cultured from E12.5 for 3 d with indicated drugs after injection of tamoxifen at E9.5 and E10.5. Scale bars: 50 μm.

Since *Smad4-null* germ cells showed severe defects in female differentiation, we speculated that the sexual fate of these PGCs may have been switched from female to male. To test this possibility, we investigated whether NANOS2, a male- specific marker, is expressed in *Smad4-null* germ cells. However, we did not observe upregulation of NANOS2 in the *Smad4*-null germ cells (S4A Fig). We reasoned that the absence of *Nanos2* gene expression may be due to RA signaling, which blocks *Nanos2* activation in the testes [4,16], and predicted that simultaneous suppression of Smad4 and RA signaling may lead to *Nanos2* induction in XX germ cells. To test this hypothesis, we collected ovaries of *Smad4 (Stella)* carrying a Cre reporter *CAG-floxed-CAT-EGFP* at E12.5 and cultured them with the RA receptor antagonist AGN193109. In this genetic background, *Smad4*-deficient cells could be detected through GFP reporter expression (Fig 5A). We found that 70% (302/431; *n* = 5) of GFP-positive (*Smad4*-null) XX germ cells expressed NANOS2 only in the presence of RAR inhibitor (Fig 5B). Importantly, neither ubiquitous deletion of *Smad4* nor RAR inhibitor treatment in control ovaries alone induced *Nanos2* expression (S4B–S4D Fig). These data suggest that Smad4 and RA signaling regulate female germ cell fate in a cooperative manner.

### Germ Cell-Specific Deletion of *Smad4* and *Stra8* Resulted in Production of Male Gonocyte-Like Cells in Ovaries

**Fig 6:**
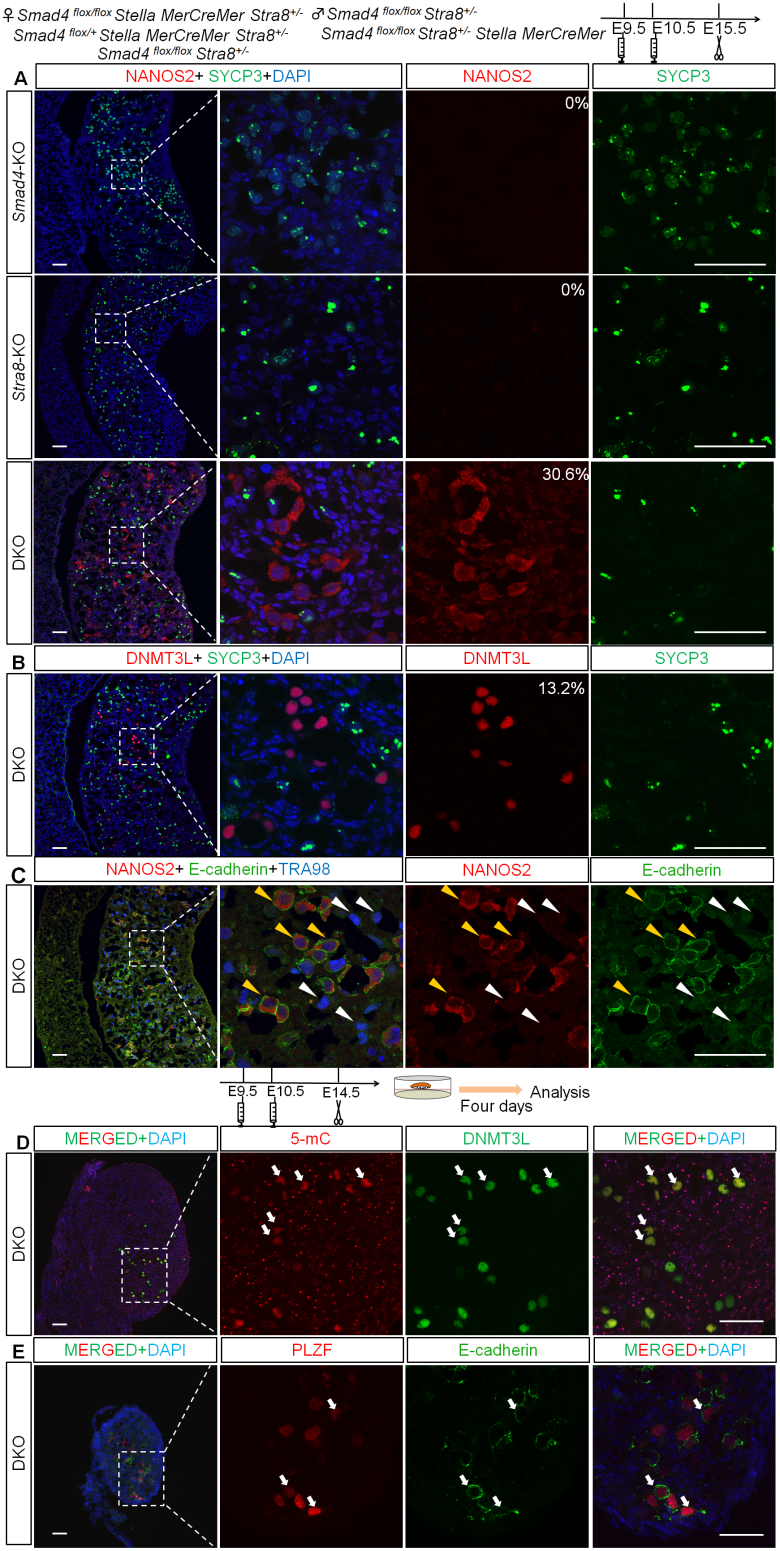
Deletion of *Smad4* and *Stra8* induces XX germ cells to adopt a male fate. (A–C) Representative image of E15.5 *Smad4*-KO (*Smad4 (Stella)*, *Stra8*^+/-^), *Stra8*-KO (*Smad4*^f/f^, *Stra8*^-/-^), and DKO ovary sections stained for SYCP3/NANOS2 (A), SYCP3/DNMT3L (B), and E-CADHERIN/NANOS2/TRA98 (C). High magnification images are shown in different channels. Numbers represent the percentage (%) of NANOS2-positive cells among SYCP3 -positive cells. Yellow arrowheads indicate NANOS2/E-CADHERIN/TRA98 triple-positive cells, and white arrowheads indicate TRA98-single positive cells. (D,E) Representative images of DKO ovary sections stained for 5-mC/DNMT3L and PLZF/E-CADHERIN. Tamoxifen was injected at E9.5 and E10.5. Gonads were cultured for 4 d from E14.5. White arrows in D and E indicate 5-mC-positive and PLZF-positive germ cells, respectively. Scale bars: 50 μm.

To test the role of Smad4 and RA signaling in female fate determination in vivo, we deleted *Stra8*, a major target of RA signaling involved in the initiation of meiosis, together with *Smad4*, and compared phenotypes among *Smad4*-null (*Smad4(Stella)/Stra8*^*+/-*^*)*, *Stra8*-null (*Smad4*^*flox/flox*^*/Stra8*^*-/-*^), and *Smad4/Stra8*-DKO (*Smad4(Stella)/Stra8*^*-/-*^*)* (referred to as DKO) germ cells. NANOS2-expressing germ cells were detected in the DKO mutants but not in *Smad4* or *Stra8* single-mutant ovaries (30.6 ± 16.5%, *n* = 4; Fig 6A). Conversely, SYCP3 expression disappeared in the NANOS2-expressing XX germ cells (Fig 6A). Intriguingly, the DKO XX germ cells also showed other male-like features: they began DNMT3L expression, a downstream factor of NANOS2 involved in de novo DNA methylation in fetal testes (13.2 ± 6.9%, n = 4; Fig 6B) [11,13], and retained E-cadherin expression (Fig 6C), a germ cell marker whose expression decreases in XX germ cells from E15.5 [46], as seen in XY germ cells (S5D and S5E Fig). These factors were never observed in *Stra8*-null ovaries (S5A and S5B Fig). These data indicate that SMAD4 and STRA8 are essential factors responsible for female fate determination.

To further trace the fate of germ cells in DKO mice, we cultured E14.5 DKO ovaries for 4 d, because all of the pregnant mice carrying DKO embryos aborted at E16.5 after TM injection. We examined two critical male gonocyte markers—5-methylcytosine (mC), a marker of DNA methylation, and promyelocytic leukemia zinc finger (PLZF) [47]—and found that most DNMT3L-positive germ cells exhibited strong 5-mC signals and some E-cadherin-positive cells had PLZF expression (Fig 6D and 6E), while no PLZF and 5-mC positive signals were detected in control ovaries (S5C Fig). These results suggest that DKO germ cells acquired an ability to enter the male gonocyte differentiation pathway.

### Female-to-Male Gene Expression Changes in DKO Germ Cells without Somatic Sex Reversal

To examine to what extent expression pattern changes in the DKO ovary, we performed microarray analyses. Total RNAs were extracted from control (*Smad4*^*flox/+*^, *Smad4*^*flox/flox*^*/Stra8*^*+/-*^) testes and ovaries and from *Smad4 (Stella)* and DKO ovaries at E14.5. To examine genes expressed in germ or somatic cells separately, we first extracted 386 and 360 genes, specifically expressed in control E13.5 male and female germ cells, as well as 680 and 636 genes, specifically expressed in control E13.5 male and female somatic cells, respectively, from our previous data (see Materials and Methods section; also see S1 and S2 Tables) [48]. The upregulation of male-specific genes in DKO ovaries showed a 1-day delay compared with normal male differentiation of germ cells in testes (for example, DNMT3L positive germ cells appeared from E14.5 in control testes but from E15.5 in DKO ovaries judging by comparing S6A Fig and Fig 6B); thus, we considered the developmental time point at E14.5 in the DKO ovary equivalent to that at E13.5 in control samples.

**Fig 7:**
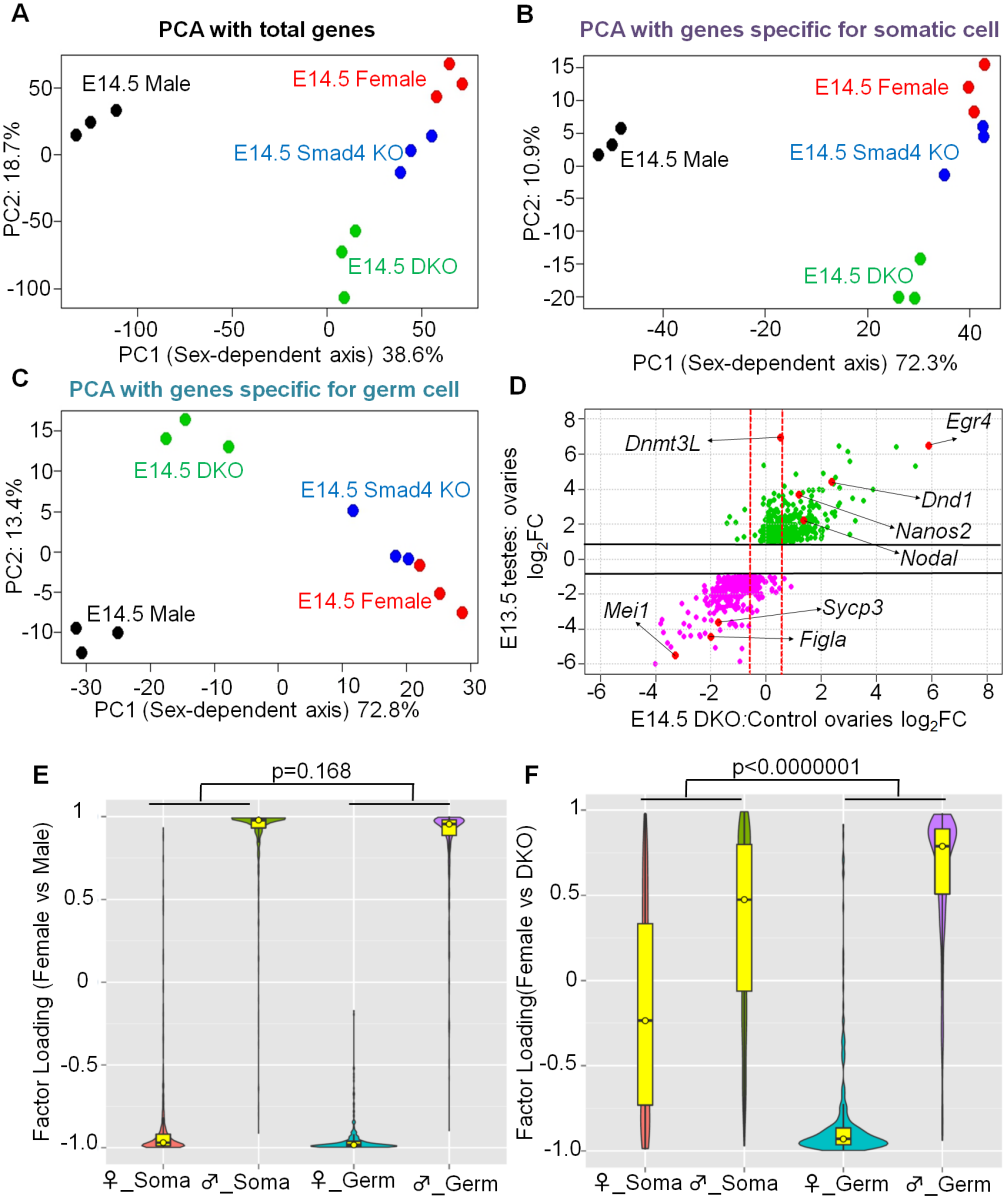
Sexual fate change of mutant XX germ cells was not accompanied by somatic sex reprogramming. (A–C) Principal component analyses of indicated samples for total genes (A), somatic-specific genes (B), and germ cell specific genes (C). (D) Scatter-plot analysis comparing germ-cell-specific gene expression changes between E13.5 Nanos3^+/−^ male and female gonads (data from [48]) and between E14.5 DKO ovaries and control ovaries. For each point (gene), the *y*-value represents the expression change comparing E13.5 testes with E13.5 ovaries and the *x*-value represents the expression change comparing E14.5 DKO ovaries and control ovaries. Thus, green and pink spots indicate 1.5-fold upregulated or downregulated expression in E13.5 testes versus ovaries (*y*-value greater than log_2_[1.5] for green spots and *y*-value less than -log_2_[1.5] for pink spots: the cut-off lines are indicated by horizontal lines). These spots are considered as genes that are specifically expressed in either male (green) or female (pink) germ cells, and their expression changes between DKO and control ovaries can be judged by the *x*-value. Red spots indicate specific gene examples. Genes located right of the rightmost dotted red line show more than a 1.5-fold increase in DKO, and genes located left of the leftmost dotted red line show less than a 1.5-fold decrease in DKO compared with control ovaries. Because genes that are specifically expressed in germ cells and somatic cells were extracted using microarray data of *Nanos3*^*+/-*^ ovaries and testes, we used *Nanos3*^*+/-*^ ovaries and testes as control. (E,F) Factor loading of indicated gene groups to principle component. PCA analysis was performed using E13.5 Nanos3^+/−^ male and female gonads (E) and E14.5 double-mutant and control ovaries (F). See text for details.

First, principal component analysis (PCA) was performed based on all genes, somatic-specific genes, and germ-specific genes, respectively (Fig 7A–7C). In all cases, PCA divided these samples into two major populations according to a putative sex-dependent axis (PC1 value = 38.6%, 72.3%, and 72.8%, respectively). Male and female control samples were located at opposite ends, as expected. When PCA was performed with all genes or somatic-specific genes, the DKO did not show a strong bias (Fig 7A and 7B). In contrast, when PCA was performed with genes specific for germ cells, the DKO samples were shifted toward the male side compared with *Smad4*-single KO samples on the putative sex-dependent axis (Fig 7C). We also observed the differences between DKO and male samples on the *y*-axis, which might be ascribed to the presence of somatic cells and the incomplete knockout efficiency. Nevertheless, it should be noted that PC1 value is as high as 72.8% in this case, suggesting that most of the gene expression profile in DKO germ cells shifts from female to male.

Next, we examined how many germ-cell-specific gene expressions were changed in DKO ovaries. Among 386 male-specific genes that showed a 1.5-fold stronger expression in E13.5 testes than in ovaries (Fig 7D, green dots with *y*-value greater than log_2_[1.5]), more than half of the cognate genes (52.1%, 201/386) were 1.5-fold upregulated in the DKO ovaries than in the control ovaries (Fig 7D, green points with *x*-value greater than log_2_[1.5]). Similarly, the expression levels of 71.1% (256/360) female-specific genes (Fig 7D, pink points whose *x*-value is less than -log_2_[1.5]) were 1.5-fold downregulated in the DKO ovaries. The results of microarray analysis were supported by RT-qPCR, in which eight out of eleven male-specific genes were upregulated in DKO ovaries (S6A Fig). Although *Dnmt3l* mRNA expression was not yet increased at E14.5, immunostaining data showed it was upregulated at E15.5 (Fig 6B). These data indicate that not only the representative genes but also a substantial number of germ-cell-specific genes were shifted to male in DKO ovaries.

The results of PCA analysis were further supported by factor loading analysis (Fig 7E and 7F). Theoretically, the genes that give a more positive contribution to the classification will have a factor loading value (*y*-axis in Fig 7E and 7F) closer to 1 or -1. In the control, the factor loading value of most of the female-specific genes (both somatic and germ cell specific) was close to -1, and that of the male-specific genes was close to 1, indicating that somatic and germ cells contribute equally to the female versus male diversification of developing gonads (see Fig 7E [*p* = 0.168]). Notably, the comparison of the DKO and control ovaries showed that the factor loading value of most of the germ-cell-specific genes, but not the somatic-cell-specific genes, was closer to 1 or -1, indicating that germ-cell-specific genes contribute much more than somatic-cell-specific genes in distinguishing DKO and control ovaries (*p* < 0.0000001; Fig 7F). Indeed, somatic cells differentiated to pregranulosa cells marked by FOXL2, but did not differentiate to Sertoli cells, as revealed by the negative signal for SOX9, in DKO ovaries (S6A and S6B Fig, also see S4D Fig). These results suggest that the observed sexual fate change of XX PGCs in DKO ovaries occurs independently from global gene expression change in the somatic cells.

## Discussion

**Fig 8:**
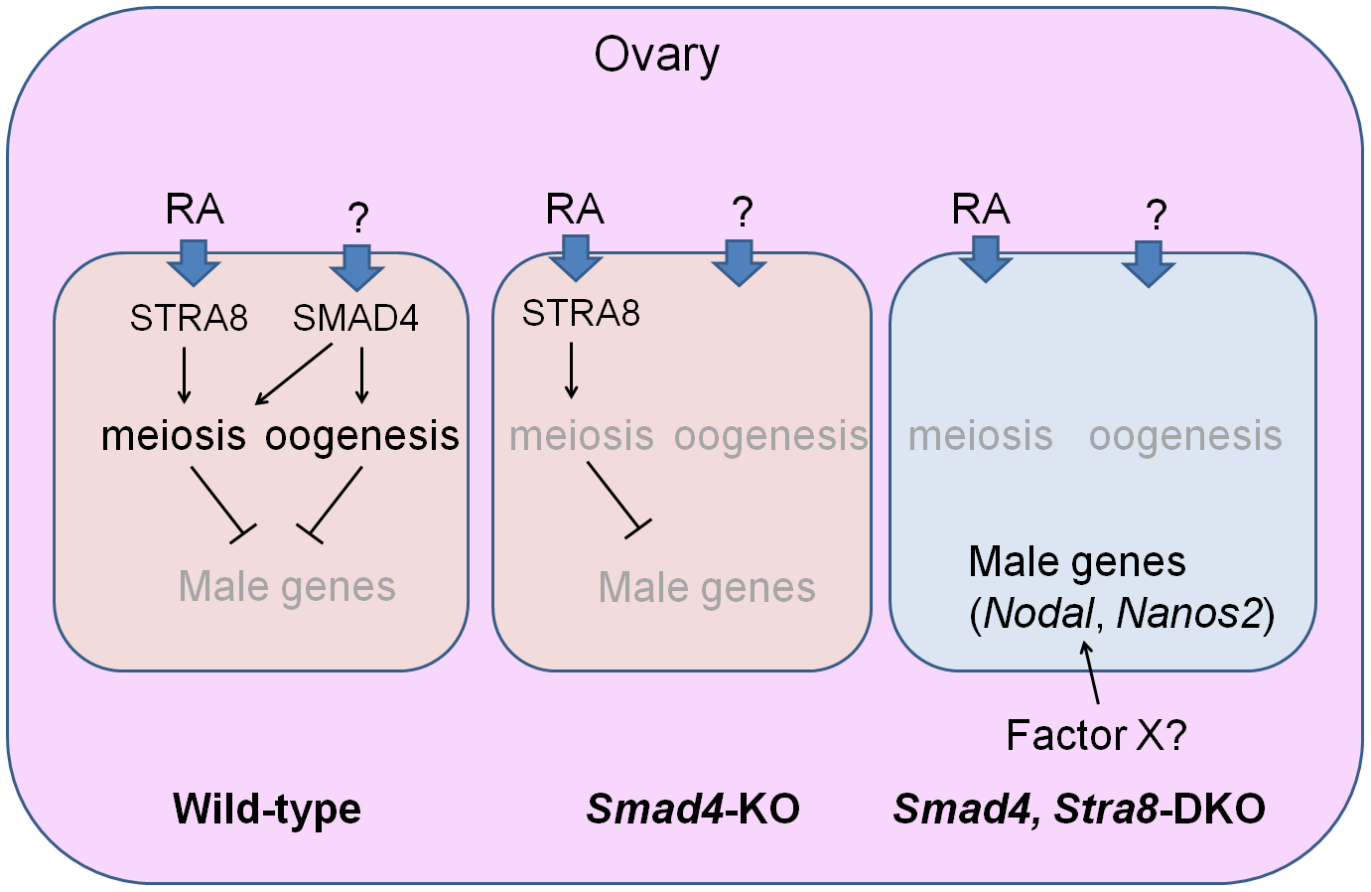
Sexual fate change in DKO germ cells in ovary. In the ovary, two signaling pathways are involved in germ cell fate determination; one is an RA-Stra8 pathway that initiates meiosis and the other is a SMAD4 pathway that is activated via a currently unknown factor. In the absence of SMAD4, germ cells fail to proceed through normal meiosis and fail to make any oocytes. SMAD4 acts in germ cells cell-autonomously and regulates the fate of female germ cells because loss of SMAD4 and STRA8 leads to the upregulation of *Nodal* and NANOS2 expression as well as other male-specific genes. In this situation, somatic cell fate was unchanged and germ cells in the DKO may determine their fate in a cell-autonomous manner or another common factor (X) from female somatic cells is also required.

In this study, we investigated the function of Smad4 signaling in the sexual differentiation of germ cells in ovaries and clarified the roles of Smad4 and RA signaling in germ cell fate determination (Fig 8). Three important outcomes in this study deserve particular attention.

First, we provided an insight into the critical question in reproductive biology: what factors determine female germ cell fate? RA-Stra8 signaling was once considered to determine female germ cell fate by inducing meiosis. However, a recent study showed a case where female germ cells can complete oogenesis even in the absence of *Stra8*, indicating that these two pathways are independent [22]. Therefore, uncovering the factors that determine female germ cell fate has been long awaited. Given that the genes involved in follicular formation were compromised in *Smad4*-deficient germ cells without affecting initiation of *Stra8* expression and knocking out of both *Smad4* and *Stra8* resulted in up-regulation of male-specific germ cell genes, we propose that SMAD4 is a strong candidate for a sexual fate determinant in female germ cells. *Lhx8* and *Figla* may be the potential targets of SMAD4, but we do not exclude that other factors may also be regulated by SMAD4. Future studies to identify the target gene(s) of SMAD4 along with functional studies would facilitate our understanding for the regulators of oogenesis. In addition, what signal SMAD4 mediates in female germ cells remains an open question. In this regard, a deduced candidate is BMP2. As BMP2 is expressed in ovarian somatic cells under the control of Wnt4 [29], we speculate that BMP2 initiates Smad4 signaling. However, we cannot exclude the possibility that other TGFβ ligands activate the pathway as well. Further analysis is required to elucidate the signaling pathway.

We conclude that Smad4 signaling in germ cells functions independent of somatic cells during the sexual fate determination of XX germ cells based on the fact that expression levels of oocyte-specific regulators in *Smad4*^*flox/flox*^*/WT1CreERT2* mutant ovaries were not significantly different from those of control ovaries at approximately E17.5. However, in the maturation stages of oocytes after birth, the deletion of *Smad4* from somatic cells (granulosa cells) is accompanied by precocious luteinization and cumulus cell defects [49]. Therefore, the function of SMAD4 is highly stage-dependent in ovaries.

Second, we found that SMAD4 or its downstream signaling regulates meiotic progression during oogenesis. Although controversial [50,51], it is widely accepted that RA induces meiosis in germ cells. However, whether RA alone supports meiotic progression or requires other signals remains unknown. RA alone could not induce meiosis in vitro, which implies that factors other than RA are involved in meiotic progression [52]. The results of our study showed that *Smad4*-null germ cells expressed *Stra8* and *Rec8*, but most showed meiotic defects before the pachytene stage accompanied by a failure to upregulate genes involved in meiosis (Fig 2 and S3B Fig). Notably, most of these genes were also inactivated in *Stra8* mutant ovaries (for example, *Sycp1*, *Sycp3*, *Dmc1*, *Spo11*, *Mei1*, and *Hormad1*) [53], which implies that SMAD4 and STRA8 regulate the same downstream factors to promote meiosis. Moreover, a Stra8-dependent pathway is reportedly responsible for the downregulation of *Stra8* and *Rec8* expression levels [53]. We speculated that this pathway is inactivated by the deletion of *Smad4* because the expression level of *Stra8* in mutant germ cells was higher than that in normal ovaries (Fig 2A and S3B Fig). Overall, the results of the present study indicated that in addition to RA-dependent meiotic initiation, Smad4 signaling is required for meiotic progression, consistent with the study that successful induction of meiosis requires Activin A, BMP, and RA [52]. This finding is a critical clue for understanding the mechanisms involved in meiosis.

Third, we found profound properties of DKO germ cells. These germ cells became positive for male factors NANOS2 and DNML3L, and some of those cells showed 5-mC, indicating DNA methylation, all of which are normally observed only in male gonocytes. The transcriptome analyses of DKO embryos showed that more than 50% of male-type genes were up-regulated and more than 70% of female type genes were downregulated, indicating that male pathway is promoted when female germ cell development is suppressed by the lack of *Stra8* and *Smad4*. However, as seen in PCA analyses, DKO germ cells were not equal to male germ cells and not all DKO germ cells expressed NANOS2. This may be due to the effects from ovarian somatic cells; some factor(s) may act to suppress or promote the male pathway. Importantly, however, we showed that the female-to-male sexual fate change of XX germ cells occurred independent of the sexual switch of somatic cells as revealed by sustained FOXL2 expression in DKO ovaries. A similar phenotype was also observed in the ubiquitous *Smad4*-cKO (via Rosa-CreERT) with an RA inhibitor (S4C and S4D Fig). Owing to the lack of a Y chromosome in XX ovaries, the induction of male-like characteristics in DKO germ cells is not linked to any somatic factors, such as FGF9 and SOX9, which are controlled by SRY. Therefore, our results raise the unexpected possibility that male-specific factors in somatic cells are unnecessary for the male fate determination of germ cells even in the testes, although we do not exclude the possibility that other somatic factors (X) existing in both testes and ovaries are involved in the induction of maleness in germ cells (Fig 8). Consistent with this hypothesis, a recent study has shown that disruption of the germ-cell-specific factor FOXL3 in XX medaka (*Oryzias latipes*) leads to male differentiation even in ovaries [54]. In the case of our mouse model, however, we cannot expect to induce functional sperm in DKO ovaries, because the deletion of *Stra8* leads to meiotic defects, and spermatogenesis requires an appropriate microenvironment supported by Sertoli cells that cannot be provided in mouse ovaries.

In summary, we analyzed the functions of SMAD4 in fetal female germ cells and found that SMAD4 is required for sexual differentiation of female germ cells. We further proved that SMAD4 and STRA8 were crucial for female sex determination, because germ cells lacking these two factors became male gonocyte-like cells, even if these germ cells were surrounded with female somatic cells. Our results provide foundational information for understanding the sex determination system in mammals, which may contribute to the goal of direct gamete induction in vitro.

## Materials and Methods

### Ethics

All mouse experiments were approved by the Animal Experimentation Committee at the National Institute of Genetics. Permission number for animal experiment is 27–13.

### Mice

Generation of floxed *Smad4* alleles and *Stra8* knockout mice has been described previously [48,55]. *Stella-MerCreMer* was established previously [33]. *Rosa-CreERT2* mice were purchased from Artemis Pharmaceuticals GmbH. *WT1-CreERT2* mice were purchased from Jackson Laboratory. *Gt(ROSA)26Sor*^*tm1Sor*^/J and *CAG-floxed-CAT-EGFP* mouse lines were established previously [56,57]. All of these mice lines were kept in a mixed background. TM was diluted in sesame oil (Nacalai Tesque) at a concentration of 10 mg/ml, and 0.5 ml of the diluted TM was injected each time. To induce recombination in *Stella-MerCreMer* mice, TM was injected at E9.5 and E10.5. To induce recombination in *Rosa-CreERT2* or *WT1-CreERT2* mice, TM was injected at E10.5 and E11.5. ICR strain mice (Clea Japan) were used as control in some cases (S1A, S5D and S5E and S6B Figs).

### Microarray and Data Analysis

RNA samples were prepared from E14.5 testes, ovaries, *Smad4* mutant ovaries, and double mutant embryos (*n* = 3 for each case). For each hybridization assay, 200 ng of total RNA was labeled with Cy3 and hybridized to a Whole Mouse Genome Oligo Microarray (G4122F; Agilent) in accordance with the manufacturer’s protocol using a Quick Amp Labeling Kit (Cat #51900424), One Color (Agilent), and Gene Expression Hybridization Kit (Agilent). Data have been deposited in Gene Expression Omnibus under accession number: GSE68773.

Extraction of genes specifically expressed in female somatic cells, male somatic cells, and male germ cells was performed using microarray data from [48] by R. To extract genes specifically expressed in male or female germ cells at E13.5, genes showing 1.5-fold changes in expression levels between *Nanos3*^*+/-*^ testes and *Nanos3*^*+/-*^ ovaries were defined as sex-specific genes. Then, genes showing 1.5-fold changes in expression levels between *Nanos3*^*+/-*^ testes and *Nanos3*^*-/-*^ testes and between *Nanos3*^*+/-*^ ovaries and *Nanos3*^*-/-*^ ovaries were defined as germ cell-specific genes, because *Nanos3*^*-/-*^ gonads (both ovaries and testes) lack germ cells at E13.5. Genes specifically expressed in male or female germ cells were obtained by the intersection of genes in the two lists. Similarly, genes specifically expressed in male or female somatic cells (if genes are not germ-cell-specific) at E13.5 were also isolated. PCA was analyzed by R using genes in the above lists.

### Organ Culture

Gonads were cultured in 24-well culture plates with DMEM containing 10% horse serum at 37°C on 5-μm nucleopore filters [58,59]. Some gonads were cultured in medium containing 4-hydroxytamoxifen (2 μM; Sigma-Aldrich), DMSO (Sigma-Aldrich), and AGN 193109 (5 μM; Toronto Research Chemical Inc.). For the experiments shown in S4B Fig, tamoxifen was injected at E10.5 and E11.5, and ovaries obtained at E12.5 were further cultured with DMSO (control) or AGN 193109 (5 μM) for 2 d or 3 d with 4-hydroxytamoxifen (2 μM) to further induce Cre-mediated *Smad4* deletion during cultivation. For the experiments shown in Fig 5, tamoxifen was injected at E9.5 and E10.5, and ovaries obtained at E12.5 were further cultured with DMSO (control) or AGN 193109 (5 μM) for 3 d with 4-hydroxytamoxifen (2 μM). For the experiments shown in Fig 6D and 6E, tamoxifen was injected at E9.5 and E10.5, and ovaries obtained at E14.5 were further cultured for 4 d.

### Reverse Transcription Real-Time Quantitative Polymerase Chain Reaction (RT-qPCR)

Total RNA was prepared from the fetal gonads of mutant embryos and the gonads of ICR strain embryos at each embryonic stage using RNeasy Mini Kits (Qiagen). Total RNA was then used for cDNA synthesis using PrimeScript RT Reagent Kits with gDNA Erase (Takara). PCR was performed with KAPA SYBR FAST qPCR Kits using a thermal cycler dice real time system (Takara). The primer pairs used for PCR amplification are listed in S3 Table.

### Histological Analysis

Histological analysis was performed as previous reported [5,8]. Briefly, gonads were fixed in 4% paraformaldehyde, embedded in OCT compound (Tissue-Tek; Sakura), and sectioned (6 μm) using a cryostat. After preincubation with 3% skim milk powder in PBST for 30 min, the sections were stained with primary antibodies: anti-TRA98 (1:10000; a gift from Y. Nishimune), anti-undifferentiated embryonic cell transcription factor 1 (1:200; Abcam), anti-SOX2 (1:200; Santa Cruz), anti-SOX9 (1:250; a gift from Y. Kanai, The University of Tokyo, Tokyo, Japan), anti-NANOS2 (1:200) [60], anti-FOXL2 (1:200; Abcam), anti-STRA8 (1:200; Abcam), anti-SCP1 (1:1000; Abcam), anti-SCP3 (1:1000; Abcam), anti-GFP (1:400; Aves labs), anti-DNMT3L (1:200; a gift from S. Yamanaka, Kyoto University, Kyoto, Japan), anti-Cleaved-Caspase-3 (1:200; Cell Signaling Technology), anti-MVH (1:400; Abcam), anti-E-Cadherin (1:100; R&D systems) anti-NOBOX (1:200; Abcam), anti-PLZF (1:200; SantaCruz), anti-5mC (1:200; Activemotife), anit-FOXO3A (1:200; Cell signaling Technology), and anti-pSMAD1/5/8 (1:200; Cell Signaling Technology). The sections were then incubated with donkey anti-rabbit/rat/goat/mouse or anti-chick IgG secondary antibodies conjugated with either Alexa 488, Alexa 594, or cy5 (1:400; Invitrogen). Primary antibodies were diluted in 3% skim milk powder in PBST. Secondary antibodies were diluted in PBST. Slides were mounted for observation under a scanning confocal microscope (Olympus FV1200 IX83). The method for LacZ staining (S1B Fig) was reported previously [61].

### Cell Counting

To trace the cell fate of *Smad4*-null germ cells in *Smad4*^*flox/flox*^/*Stella-MerCreMer* ovaries, tamoxifen was injected at E9.5 and E10.5, and samples were harvested from E14.5 to E17.5. Ovaries were sectioned and immunostained with different markers: NOBOX (Fig 3B), FOXO3A (Fig 3C), and Cleaved-Caspase3 (Fig 1D), together with germ cell markers TRA98 or MVH. Germ cells on all these sections were counted as TRA98- or MVH-positive cells (Fig 1C). Underlying data used for counting were presented in S1 Data.

### Statistical Analysis

Statistical analysis was performed as previous reported [5,8]. For quantitative analyses between two different samples, significance was assessed by Student’s *t* test. For quantitative analyses among multiple samples, significance was assessed using one-way ANOVA followed by Tukey’s post-hoc tests for selected pairs of genotypes.

## Supporting Information

S1 Data Underlying data used for quantitative analysis. (XLSX)

S1 Fig Examination of pSMAD1/5/8 expression and Stella-MerCreMer activity in embryonic ovaries. (A) Immunohistochemical detection of pSMAD1/5/8 and TRA98 (a germ cell marker) in indicated ovaries. White arrows indicated germ cells. (B) X-gal staining followed by TRA98 immunostaining of ovary section of ROSA26-Cre reporter strain, *Gt(ROSA)26Sor*^*tm1Sor*^/J) crossed with a *Stella-MerCreMer* male mouse. Tamoxifen was injected at E9.5 and E10.5, and ovaries harvested at E14.5 were subjected to X-gal staining.
(TIF)

S2 Fig Smad4 signaling in somatic cells is not involved in sex determination of germ cells. (A) Scheme of somatic-cell-specific knockout strategy. Tamoxifen was injected at E10.5 and E11.5, and ovaries were harvested at E14.5 (B) or cultured for 3 d (C). (B) Expression levels of the indicated genes were compared by RT-qPCR in control (*n* = 6) and *Smad4* mutant (via WT1-CreERT2; briefly WT1) ovaries (*n* = 4). The expression levels of the indicated genes were normalized to that of *Mvh* or *G3phd* (for *Smad4*). (C) Quantitative analysis of NOBOX-positive germ cells in *Smad4* mutant (WT1) (*n* = 3) and control ovaries (*n* = 4). Significance was assessed by Student’s *t* test. Error bars indicate SD. Underlying data is available in S1 Data.
(TIF)

S3 Fig Ubiquitous deletion of *Smad4* reproduced the phenotype of germ-line-specific *Smad4* deletion in ovaries. (A) Schematic drawing of ubiquitous knockout strategy. Tamoxifen was injected at E10.5 and E11.5, and ovaries were harvested at E14.5. (B) Expression level of meiosis-related genes in control and *Smad4* mutant (Rosa) ovaries. Data are represented as a heat map and the *p*-value was calculated by Student’s *t* test. (C,D) Expression levels of the indicated genes were compared by RT-qPCR in control (set as 1) and *Smad4* mutant (Rosa) ovaries (*n* = 4). The expression levels of the indicated genes were normalized to that of mouse vasa homolog (*Mvh*). Significance was assessed using Student’s *t* test for one pair of genotypes and one-way ANOVA followed by Tukey’s post-hoc tests for selected pairs of genotypes. Error bars indicate SD. Underlying data is available in S1 Data.
(TIF)

S4 Fig Suppression of Smad4 or RA signaling alone does not result in sex reversal of XX PGCs. (A) Expression level of indicated genes in control and *Smad4 (Stella)* ovaries at E14.5. Data was extracted from microarray analysis. (B) Experimental scheme for (C,D). (C) RT-qPCR analysis of *Stra8* and *Nanos2* expression in control and *Smad4*-cKO (Rosa) mutant ovaries that were or were not treated with the RA receptor antagonist AGN 193109 for 2 d. Error bars indicate SD. Underlying data is available in S1 Data. (D) Representative image of sections from *Smad4*-cKO (Rosa) ovaries stained for NANOS2 and FOXL2 after treatment with AGN or DMSO for 3 d. Scale bar: 50 μm.
(TIF)

S5 Fig Induction of male-specific genes was not observed in either *Stra8*-KO or wild-type ovaries. (A,B) Representative images of *Stra8-KO* ovary sections (littermate control of Fig 6B and 6C). (C) Representative images of wild-type ovaries incubated for 4 d with normal medium and stained for 5-mC and PLZF (negative control). (D,E) Wild-type testes stained for E-CADHERIN, PLZF, DNMT3L, and 5-mC, related to Fig 6D and 6E. Scale bars: 50 μm.
(TIF)

S6 Fig Induction of male-specific genes occurred independently of sex reversal of somatic cells in DKO ovaries. (A) Expression levels of the indicated genes were compared by RT-qPCR in control male (set as 1) and female gonads, and in double mutant ovaries (*n* = 3). Tamoxifen was injected at E9.5 and E10.5, and gonads were recovered at E14.5. The expression levels of the indicated genes were normalized to that of mouse vasa homolog (*Mvh*). Significance was assessed using Student’s *t* test. Error bars indicate SD. Underlying data is available in S1 Data. (B) Representative image of E15.5 DKO ovarian tissue section stained for FOXL2 and SOX9, and wild-type testis section stained for SOX9 and TRA98. Scale bars: 50 μm.
(TIF)

S1 Table Fold change of male germ-cell-specific genes in DKO ovaries compared with control ovaries. (DOCX)

S2 Table Fold change of female germ-cell-specific genes in DKO ovaries compared with control ovaries. (DOCX)

S3 Table Primer set used for RT-qPCR. (DOCX)

## Abbreviations

5mC: 5-methylcytosine
BMP: bone morphogenetic protein
DNMT3L: DNA methyltransferase 3-like protein
FGF9: fibroblast growth factor 9
FOXO3a: Forkhead box O3a
NOBOX: newborn ovary homeobox protein
OCT4: octamer-binding transcription factor 4
PCA: principal component analysis
PGC: primordial germ cell
PLZF: promyelocytic leukemia zinc finger
pSMAD: phosphorylated SMAD
RA: retinoic acid
SC: synaptonemal complex
SYCP: synaptonemal complex protein
SOX: SRY-box
SRY: sex-determining region Y
SMAD4: mothers against decapentaplegic homolog 4
STRA8: stimulated by retinoic acid 8
TGFβ: transforming growth factor β
TM: tamoxifen
UTF1: undifferentiated embryonic cell transcription factor 1
WNT4: wingless-related MMTV integration site 4
