# Supplementary figures and images for: Sexual Fate Change of XX Germ Cells Caused by the Deletion of SMAD4 and STRA8 Independent of Somatic Sex Reprogramming
Source: PLoS Biol. 2016 Sep 8;14(9):e1002553. doi: 10.1371/journal.pbio.1002553 (PMC5015973; doi:10.1371/journal.pbio.1002553)

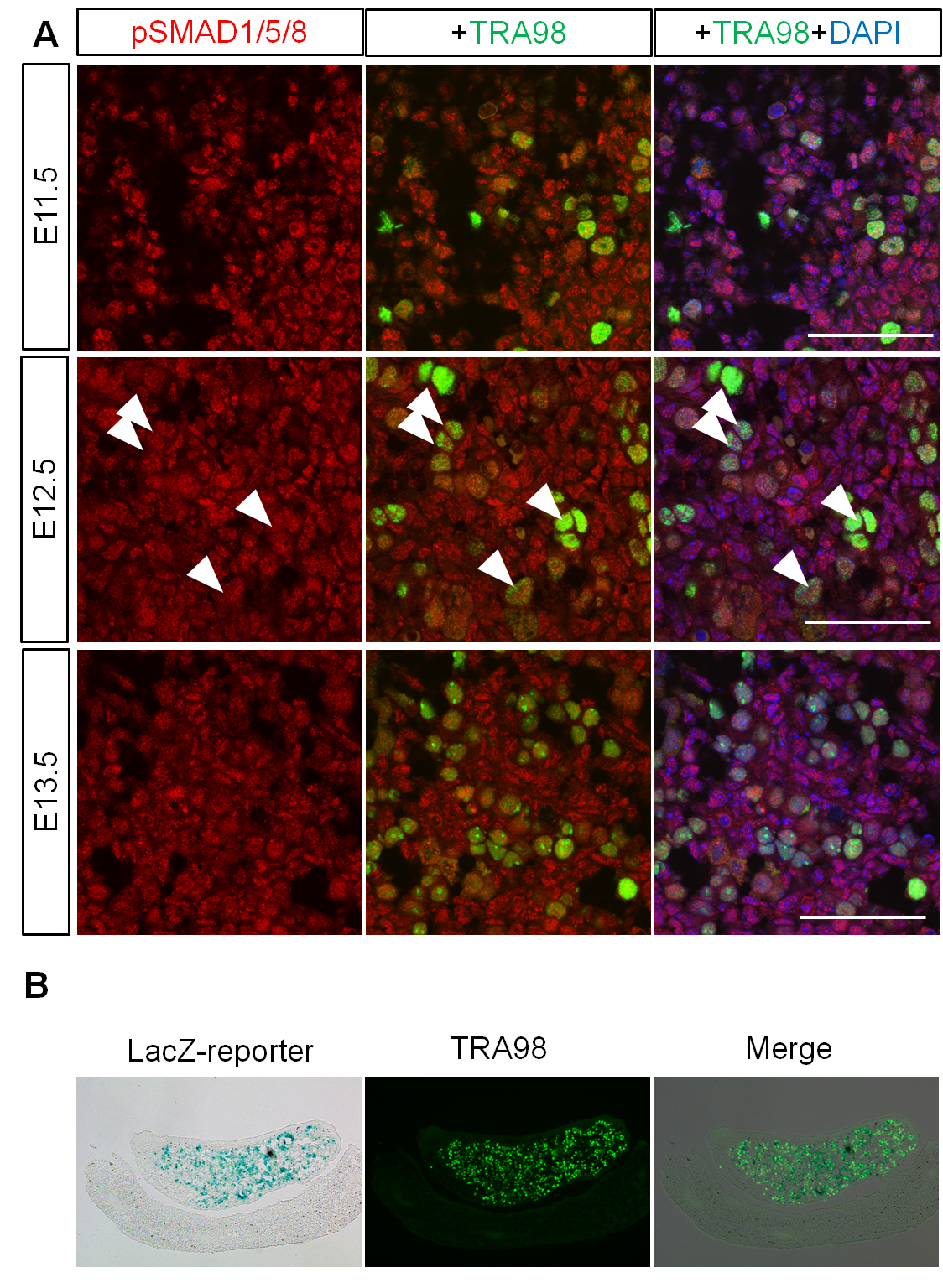

Supplement: S1 Fig — (A) Immunohistochemical detection of pSMAD1/5/8 and TRA98 (a germ cell marker) in indicated ovaries. White arrows indicated germ cells. (B) X-gal staining followed by TRA98 immunostaining of ovary section of ROSA26-Cre reporter strain, Gt(ROSA)26Sortm1Sor/J) crossed with a Stella-MerCreMer male mouse. Tamoxifen was injected at E9.5 and E10.5, and ovaries harvested at E14.5 were subjected to X-gal staining. (TIF) [file pbio.1002553.s002.tif]

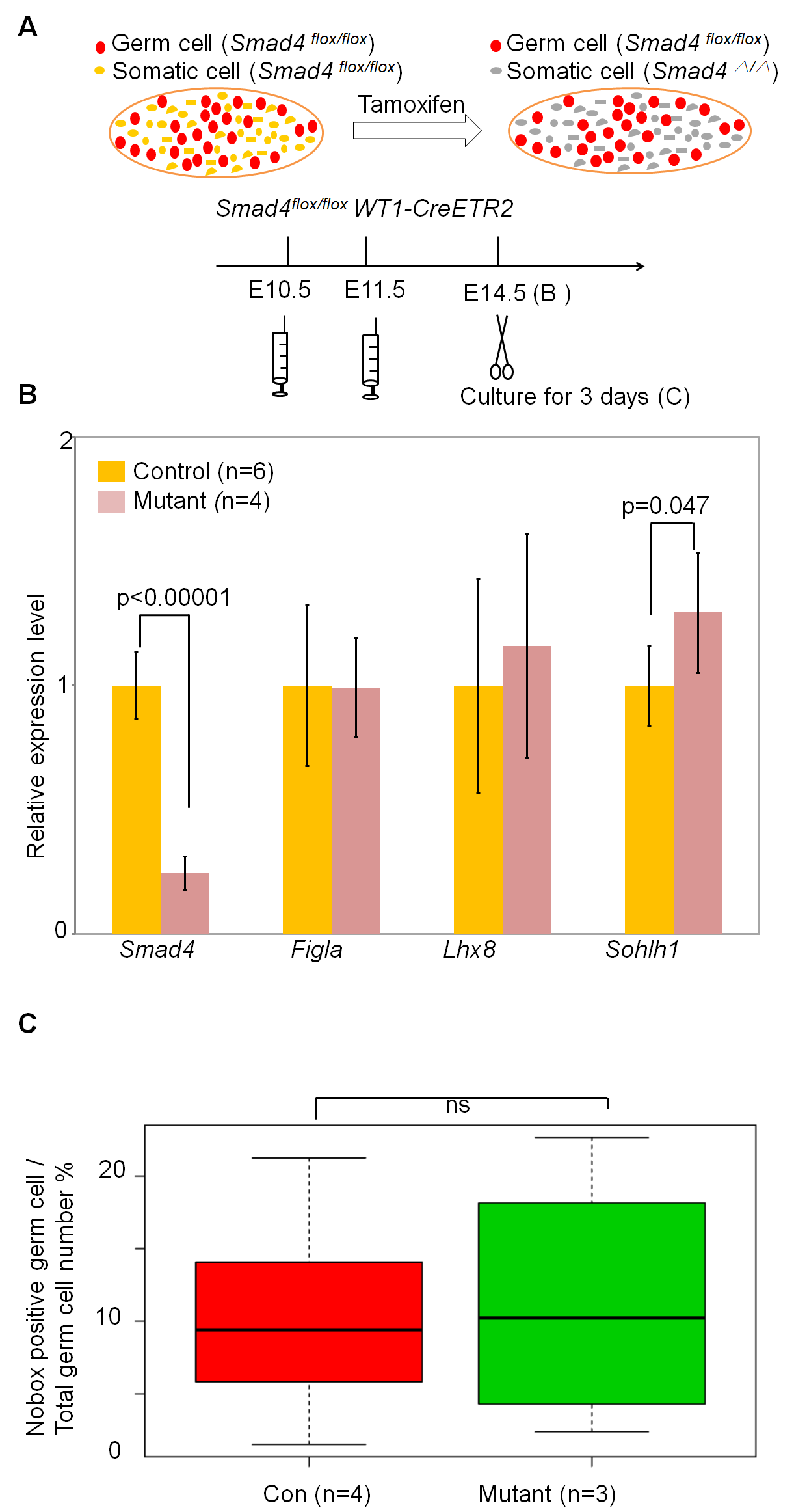

Supplement: S2 Fig — (A) Scheme of somatic-cell-specific knockout strategy. Tamoxifen was injected at E10.5 and E11.5, and ovaries were harvested at E14.5 (B) or cultured for 3 d (C). (B) Expression levels of the indicated genes were compared by RT-qPCR in control (n = 6) and Smad4 mutant (via WT1-CreERT2; briefly WT1) ovaries (n = 4). The expression levels of the indicated genes were normalized to that of Mvh or G3phd (for Smad4). (C) Quantitative analysis of NOBOX-positive germ cells in Smad4 mutant (WT1) (n = 3) and control ovaries (n = 4). Significance was assessed by Student’s t test. Error bars indicate SD. Underlying data is available in S1 Data. (TIF) [file pbio.1002553.s003.tif]

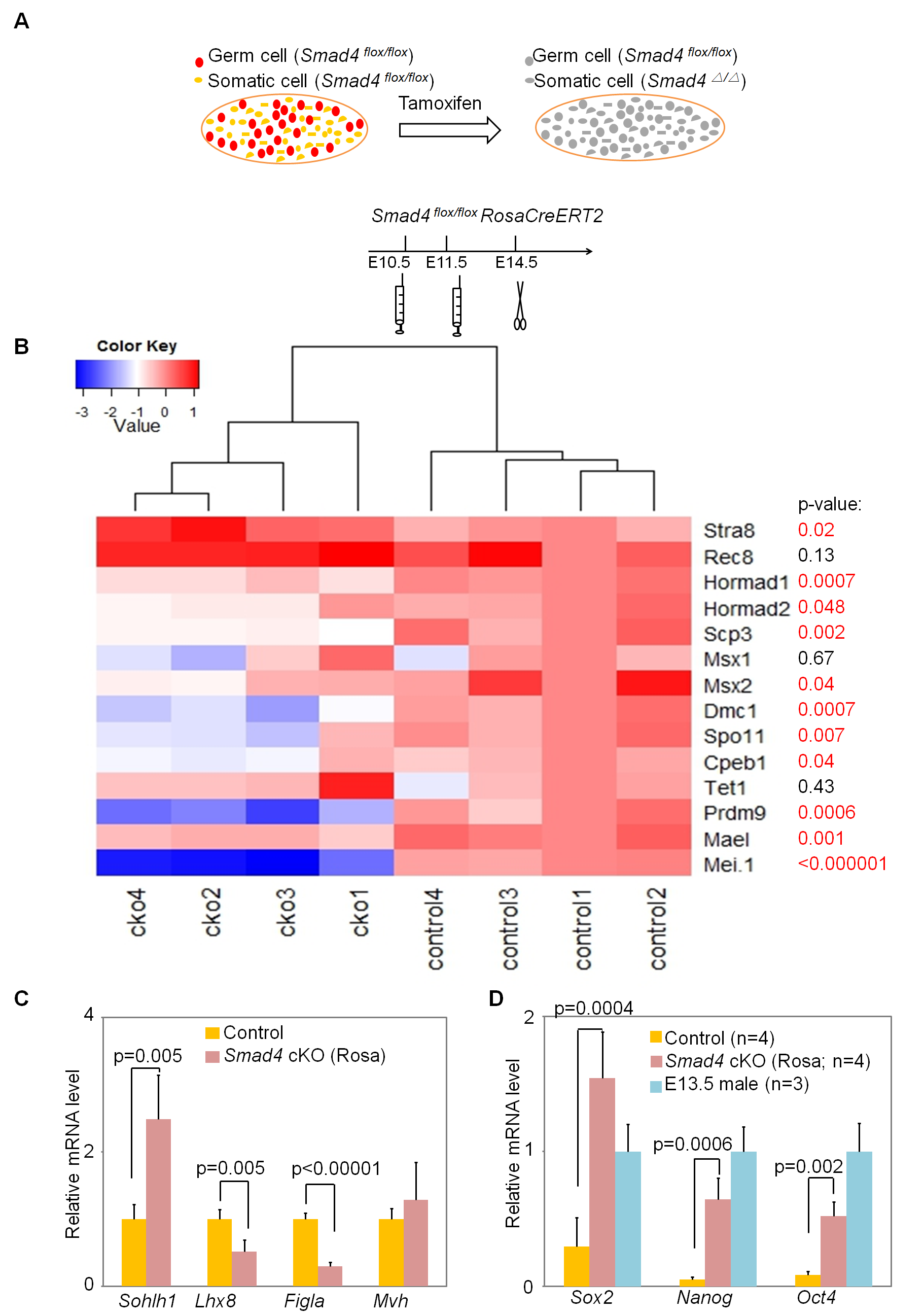

Supplement: S3 Fig — (A) Schematic drawing of ubiquitous knockout strategy. Tamoxifen was injected at E10.5 and E11.5, and ovaries were harvested at E14.5. (B) Expression level of meiosis-related genes in control and Smad4 mutant (Rosa) ovaries. Data are represented as a heat map and the p-value was calculated by Student’s t test. (C,D) Expression levels of the indicated genes were compared by RT-qPCR in control (set as 1) and Smad4 mutant (Rosa) ovaries (n = 4). The expression levels of the indicated genes were normalized to that of mouse vasa homolog (Mvh). Significance was assessed using Student’s t test for one pair of genotypes and one-way ANOVA followed by Tukey’s post-hoc tests for selected pairs of genotypes. Error bars indicate SD. Underlying data is available in S1 Data. (TIF) [file pbio.1002553.s004.tif]

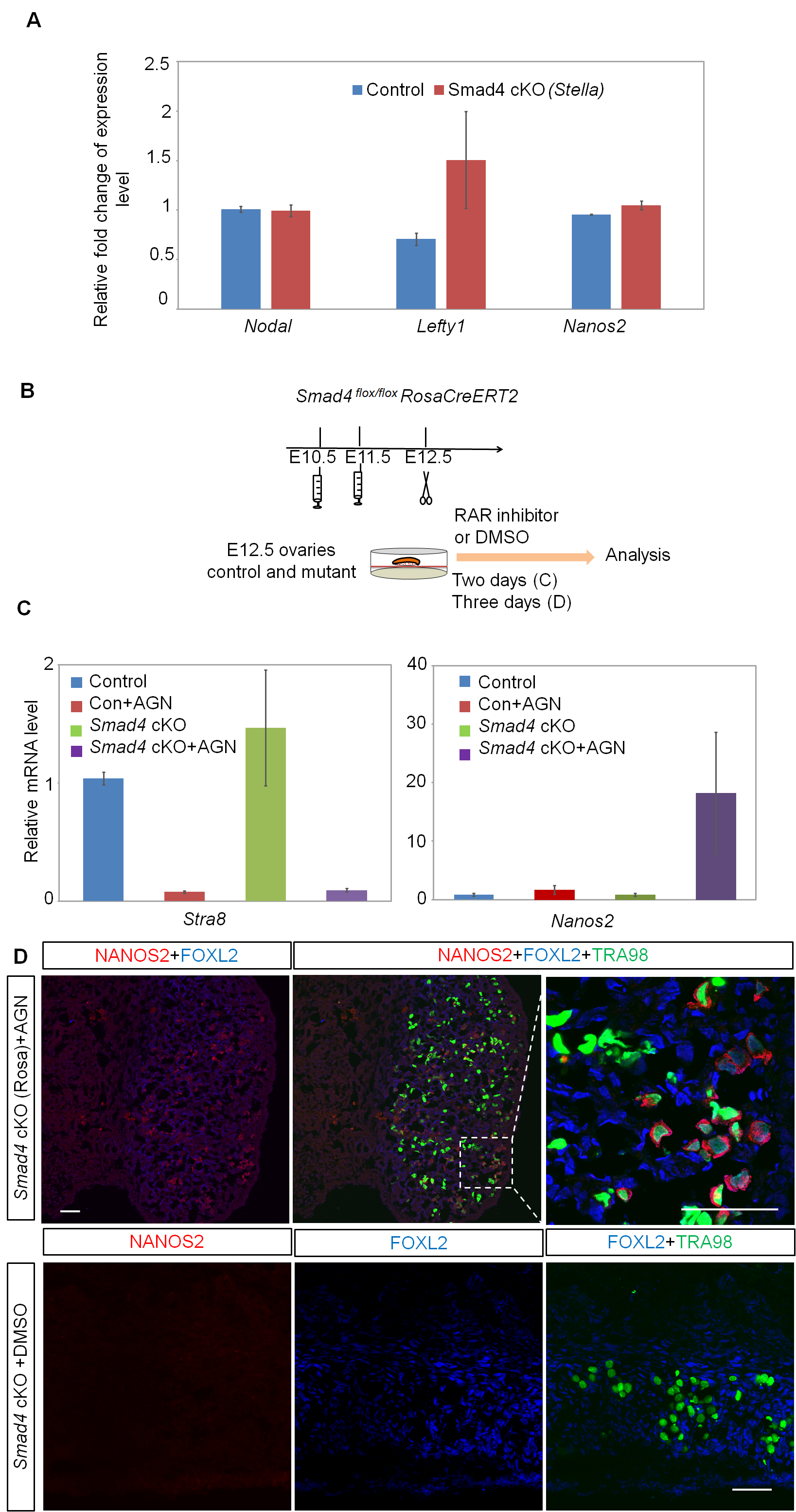

Supplement: S4 Fig — (A) Expression level of indicated genes in control and Smad4 (Stella) ovaries at E14.5. Data was extracted from microarray analysis. (B) Experimental scheme for (C,D). (C) RT-qPCR analysis of Stra8 and Nanos2 expression in control and Smad4-cKO (Rosa) mutant ovaries that were or were not treated with the RA receptor antagonist AGN 193109 for 2 d. Error bars indicate SD. Underlying data is available in S1 Data. (D) Representative image of sections from Smad4-cKO (Rosa) ovaries stained for NANOS2 and FOXL2 after treatment with AGN or DMSO for 3 d. Scale bar: 50 μm. (TIF) [file pbio.1002553.s005.tif]

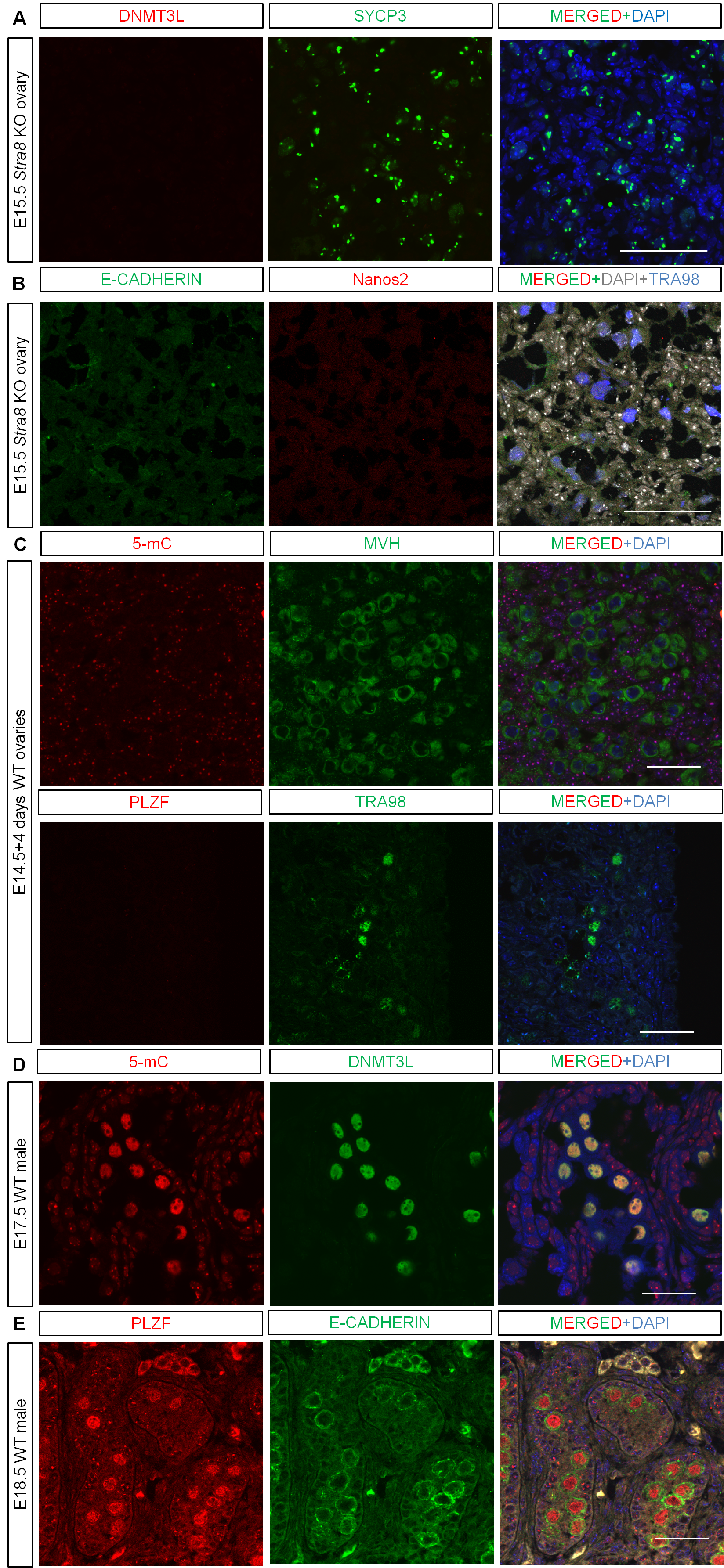

Supplement: S5 Fig — (A,B) Representative images of Stra8-KO ovary sections (littermate control of Fig 6B and 6C). (C) Representative images of wild-type ovaries incubated for 4 d with normal medium and stained for 5-mC and PLZF (negative control). (D,E) Wild-type testes stained for E-CADHERIN, PLZF, DNMT3L, and 5-mC, related to Fig 6D and 6E. Scale bars: 50 μm. (TIF) [file pbio.1002553.s006.tif]

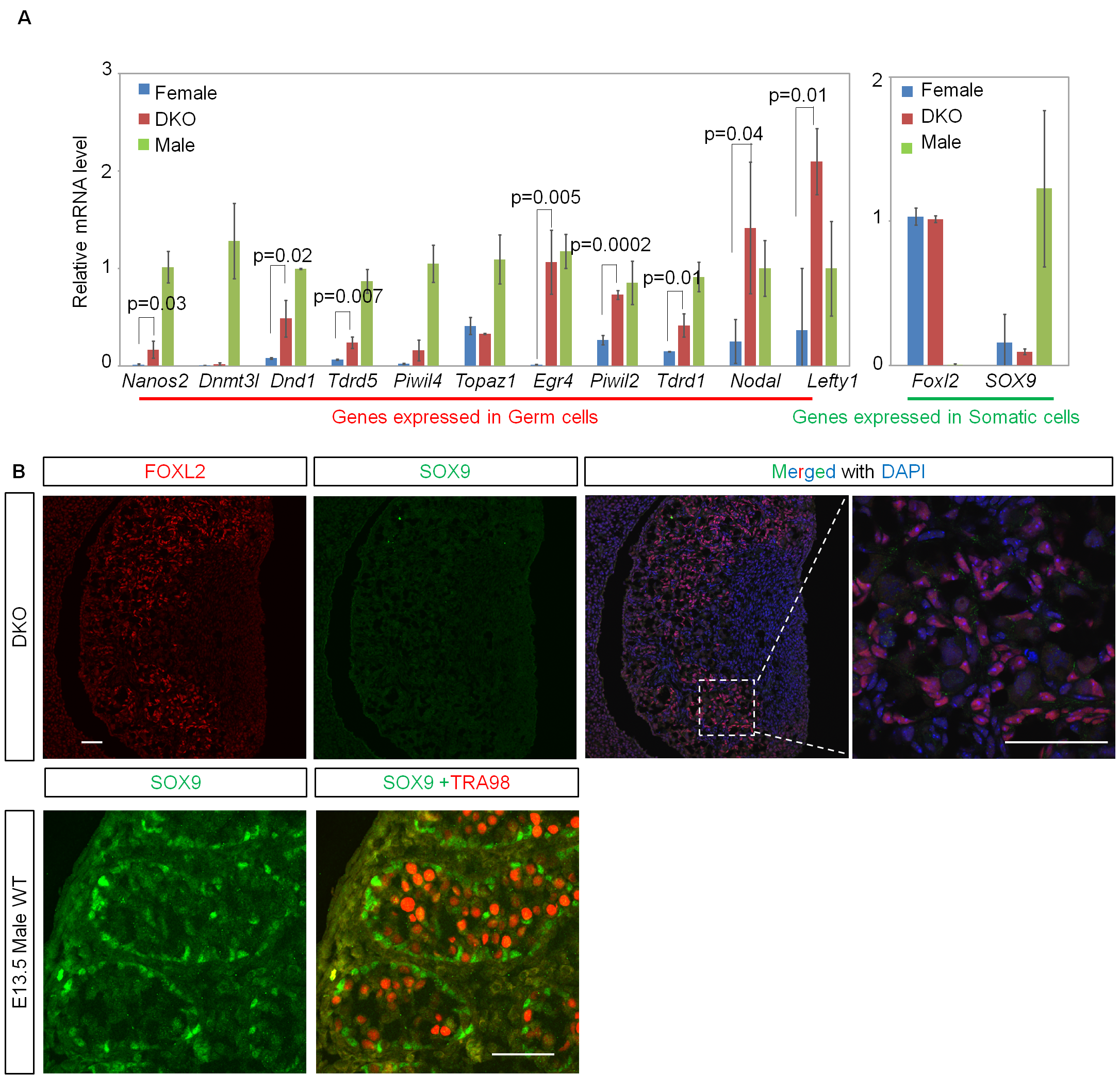

Supplement: S6 Fig — (A) Expression levels of the indicated genes were compared by RT-qPCR in control male (set as 1) and female gonads, and in double mutant ovaries (n = 3). Tamoxifen was injected at E9.5 and E10.5, and gonads were recovered at E14.5. The expression levels of the indicated genes were normalized to that of mouse vasa homolog (Mvh). Significance was assessed using Student’s t test. Error bars indicate SD. Underlying data is available in S1 Data. (B) Representative image of E15.5 DKO ovarian tissue section stained for FOXL2 and SOX9, and wild-type testis section stained for SOX9 and TRA98. Scale bars: 50 μm. (TIF) [file pbio.1002553.s007.tif]
