# Supplementary material for: Sexual Fate Change of XX Germ Cells Caused by the Deletion of SMAD4 and STRA8 Independent of Somatic Sex Reprogramming
Source: PLoS Biol. 2016 Sep 8;14(9):e1002553. doi: 10.1371/journal.pbio.1002553 (PMC5015973; doi:10.1371/journal.pbio.1002553)
Supplement: S1 Table — (DOCX) [file pbio.1002553.s008.docx]

| ID | logFC | AveExpr | P.Value | adj.P.Val |
| --- | --- | --- | --- | --- |
| Egr4 | 5.916333 | 11.2895 | 2.88E-14 | 3.43E-14 |
| Asb9 | 5.402 | 9.620417 | 2.74E-12 | 2.82E-12 |
| Pramef12 | 4.714333 | 11.12342 | 7.66E-16 | 1.32E-15 |
| Nlrp4c | 3.866333 | 10.23975 | 4.16E-14 | 4.78E-14 |
| Slc16a11 | 3.534333 | 9.758667 | 4.90E-12 | 5.02E-12 |
| Khdc1a | 3.24 | 9.216083 | 8.37E-14 | 9.15E-14 |
| Wdr16 | 3.146667 | 8.640167 | 5.19E-12 | 5.30E-12 |
| Ccno | 3.039667 | 8.571667 | 3.36E-14 | 3.92E-14 |
| Gtsf1l | 3.031667 | 8.318417 | 6.00E-13 | 6.24E-13 |
| Dppa4 | 2.972333 | 10.78708 | 2.56E-13 | 2.71E-13 |
| Nxf2 | 2.675333 | 9.229417 | 2.14E-15 | 3.25E-15 |
| Fthl17 | 2.658667 | 8.317083 | 8.27E-15 | 1.07E-14 |
| Piwil4 | 2.651667 | 10.28125 | 1.64E-14 | 2.01E-14 |
| Nlrp14 | 2.620333 | 8.301833 | 2.77E-16 | 6.09E-16 |
| Morc1 | 2.512 | 9.714167 | 1.20E-15 | 1.94E-15 |
| Uchl1 | 2.486 | 12.16933 | 1.82E-16 | 4.63E-16 |
| 1700019A02Rik | 2.484333 | 8.527083 | 7.95E-15 | 1.03E-14 |
| Dppa3 | 2.430667 | 13.79267 | 5.20E-15 | 7.05E-15 |
| Cpsf4l | 2.393333 | 9.330917 | 3.18E-12 | 3.26E-12 |
| Dnd1 | 2.393 | 10.98142 | 3.12E-14 | 3.69E-14 |
| Cdh1 | 2.215333 | 8.5855 | 1.06E-14 | 1.34E-14 |
| Tex19.2 | 2.213667 | 11.19675 | 3.07E-16 | 6.54E-16 |
| Shcbp1l | 2.151 | 8.922083 | 4.02E-15 | 5.62E-15 |
| Pdzk1 | 2.078667 | 8.103583 | 3.78E-13 | 3.96E-13 |
| Pou5f1 | 2.051667 | 9.975833 | 2.96E-14 | 3.51E-14 |
| Themis2 | 2.037333 | 8.956083 | 5.78E-14 | 6.43E-14 |
| 4930503E14Rik | 2.029667 | 7.4155 | 2.32E-14 | 2.81E-14 |
| Mageb4 | 2.024333 | 11.64117 | 3.27E-17 | 1.58E-16 |
| Gm9 | 2.018667 | 8.946667 | 5.65E-14 | 6.30E-14 |
| Tdrd12 | 1.9885 | 9.827417 | 9.37E-15 | 1.20E-14 |
| Sox2 | 1.960667 | 9.61325 | 9.46E-13 | 9.79E-13 |
| Mbl2 | 1.935333 | 8.801167 | 1.21E-15 | 1.94E-15 |
| Sult4a1 | 1.928333 | 8.501042 | 1.53E-14 | 1.90E-14 |
| Foxg1 | 1.837333 | 7.4795 | 2.83E-15 | 4.11E-15 |
| Liph | 1.814167 | 7.97475 | 1.05E-13 | 1.14E-13 |
| Nanog | 1.792 | 7.37375 | 1.18E-14 | 1.48E-14 |
| Slc4a5 | 1.786333 | 8.788917 | 1.42E-16 | 3.97E-16 |
| Rnf125 | 1.777333 | 8.23025 | 6.64E-15 | 8.74E-15 |
| Tmc7 | 1.764667 | 8.86375 | 5.94E-16 | 1.07E-15 |
| Rimkla | 1.750667 | 9.163833 | 2.41E-15 | 3.57E-15 |
| Alpl | 1.711 | 11.97125 | 4.04E-14 | 4.66E-14 |
| Utf1 | 1.706333 | 8.5145 | 1.32E-13 | 1.43E-13 |
| Hrh1 | 1.7 | 7.337583 | 2.67E-13 | 2.81E-13 |
| Fam183b | 1.699667 | 7.62575 | 6.14E-14 | 6.81E-14 |
| Hemt1 | 1.681667 | 8.172833 | 8.46E-16 | 1.43E-15 |
| Cpm | 1.66 | 9.402333 | 3.65E-15 | 5.17E-15 |
| Mlana | 1.645333 | 8.46175 | 6.38E-16 | 1.13E-15 |
| Blnk | 1.626667 | 9.465667 | 3.83E-13 | 4.00E-13 |
| Zfp819 | 1.614 | 8.4675 | 1.58E-14 | 1.95E-14 |
| AI854703 | 1.598 | 8.672583 | 6.12E-12 | 6.22E-12 |
| Kif5a | 1.592667 | 9.091417 | 4.90E-14 | 5.58E-14 |
| Nanos3 | 1.58 | 7.465 | 2.65E-14 | 3.17E-14 |
| Adad2 | 1.575833 | 9.028792 | 6.83E-15 | 8.97E-15 |
| Krt222 | 1.572333 | 7.482917 | 7.33E-15 | 9.59E-15 |
| Atp6v1c2 | 1.565667 | 8.0825 | 1.49E-13 | 1.61E-13 |
| 2410018M08Rik | 1.558333 | 9.429333 | 2.01E-16 | 5.01E-16 |
| Vstm2l | 1.548333 | 8.923667 | 3.26E-16 | 6.77E-16 |
| Abcc4 | 1.536 | 10.38033 | 1.67E-13 | 1.79E-13 |
| Psma8 | 1.534333 | 10.96517 | 4.75E-16 | 9.03E-16 |
| Magea8 | 1.514 | 7.567333 | 2.33E-14 | 2.81E-14 |
| Dydc2 | 1.508667 | 8.476333 | 5.44E-14 | 6.11E-14 |
| Slc35f2 | 1.475667 | 8.57675 | 4.80E-15 | 6.57E-15 |
| Map3k15 | 1.473 | 9.098333 | 2.06E-16 | 5.05E-16 |
| Acot10 | 1.468333 | 10.23258 | 4.28E-16 | 8.26E-16 |
| Kcnj4 | 1.465333 | 8.669417 | 3.80E-14 | 4.39E-14 |
| 1700049E17Rik1 | 1.450333 | 7.561333 | 5.60E-12 | 5.71E-12 |
| Tmem40 | 1.416333 | 8.352583 | 1.22E-15 | 1.96E-15 |
| Rpl39l | 1.412 | 10.86583 | 1.89E-17 | 1.32E-16 |
| Glis1 | 1.38 | 9.040083 | 1.40E-14 | 1.75E-14 |
| Rrp9 | 1.371 | 9.625 | 2.81E-16 | 6.13E-16 |
| Nodal | 1.367 | 7.586917 | 3.61E-14 | 4.18E-14 |
| Zfp640 | 1.365417 | 8.967896 | 1.50E-15 | 2.33E-15 |
| Tcl1 | 1.352667 | 7.936333 | 3.35E-14 | 3.92E-14 |
| Padi3 | 1.334667 | 9.257167 | 4.81E-14 | 5.50E-14 |
| Plcg2 | 1.334333 | 11.44175 | 2.46E-15 | 3.63E-15 |
| Rbks | 1.327333 | 10.13458 | 2.18E-15 | 3.30E-15 |
| Usp28 | 1.324667 | 11.158 | 1.16E-19 | 1.09E-17 |
| Plk5 | 1.303333 | 8.571917 | 5.26E-14 | 5.96E-14 |
| Mageb16 | 1.290333 | 9.7755 | 7.67E-16 | 1.32E-15 |
| Nphp4 | 1.287667 | 9.495417 | 1.17E-15 | 1.90E-15 |
| Camk2b | 1.283333 | 7.874333 | 7.80E-15 | 1.02E-14 |
| Trim13 | 1.265 | 9.381583 | 2.42E-15 | 3.59E-15 |
| Fam163a | 1.263667 | 7.495083 | 1.26E-15 | 2.02E-15 |
| Slc32a1 | 1.258333 | 7.36325 | 4.50E-13 | 4.69E-13 |
| Acot9 | 1.257333 | 11.00817 | 1.49E-16 | 4.10E-16 |
| Rsph9 | 1.252 | 11.05608 | 1.42E-17 | 1.21E-16 |
| Ctrc | 1.251333 | 8.233583 | 1.84E-15 | 2.80E-15 |
| Spint1 | 1.2495 | 8.408542 | 3.97E-15 | 5.59E-15 |
| 2410012M07Rik | 1.247 | 7.576917 | 9.14E-15 | 1.17E-14 |
| Hspbap1 | 1.231667 | 10.70458 | 2.39E-14 | 2.87E-14 |
| 2010300C02Rik | 1.2255 | 8.963917 | 3.77E-17 | 1.68E-16 |
| Piwil2 | 1.221333 | 10.2885 | 2.97E-16 | 6.38E-16 |
| Pde4c | 1.214667 | 6.974917 | 1.78E-13 | 1.90E-13 |
| Nanos2 | 1.199333 | 7.431667 | 5.23E-15 | 7.05E-15 |
| 2410141K09Rik | 1.197667 | 8.823333 | 8.60E-14 | 9.38E-14 |
| Fhl1 | 1.197 | 13.19642 | 2.05E-17 | 1.33E-16 |
| Pmm1 | 1.190167 | 11.93725 | 6.04E-17 | 2.16E-16 |
| Ces1c | 1.17 | 7.2015 | 4.80E-14 | 5.50E-14 |
| Six1 | 1.162222 | 7.775944 | 2.89E-14 | 3.43E-14 |
| Sdc4 | 1.157833 | 10.00854 | 8.86E-17 | 2.85E-16 |
| Kremen2 | 1.154667 | 7.591167 | 4.22E-15 | 5.86E-15 |
| Rab11fip1 | 1.123667 | 8.77075 | 2.85E-17 | 1.46E-16 |
| Pkp2 | 1.120667 | 10.94967 | 1.62E-16 | 4.37E-16 |
| Lin28a | 1.114667 | 8.10125 | 7.39E-14 | 8.10E-14 |
| Sall4 | 1.1135 | 8.141208 | 1.62E-15 | 2.51E-15 |
| 9630033F20Rik | 1.11 | 8.662333 | 3.32E-14 | 3.90E-14 |
| Tdrd1 | 1.0905 | 9.634333 | 3.94E-16 | 7.81E-16 |
| Rhox5 | 1.088 | 14.43208 | 3.68E-16 | 7.43E-16 |
| Pik3cd | 1.082667 | 10.09617 | 3.54E-15 | 5.04E-15 |
| Dppa2 | 1.081333 | 7.863417 | 9.58E-15 | 1.22E-14 |
| Dbx2 | 1.08 | 7.729333 | 1.62E-14 | 1.99E-14 |
| Acp6 | 1.078333 | 12.2585 | 1.00E-18 | 2.77E-17 |
| Tmem217 | 1.067333 | 7.397917 | 1.06E-13 | 1.15E-13 |
| Rhebl1 | 1.064333 | 9.9775 | 1.06E-16 | 3.14E-16 |
| Ndrg4 | 1.0635 | 8.646708 | 3.01E-18 | 6.11E-17 |
| Dbndd2 | 1.063 | 10.15758 | 3.01E-17 | 1.49E-16 |
| Wnt3 | 1.060167 | 7.428625 | 1.45E-15 | 2.28E-15 |
| Clec10a | 1.042333 | 9.6465 | 9.30E-13 | 9.65E-13 |
| Mapk10 | 1.030667 | 7.787083 | 1.15E-15 | 1.88E-15 |
| Bend4 | 1.004333 | 10.98308 | 5.62E-15 | 7.53E-15 |
| Enkur | 1.001667 | 7.795667 | 2.29E-16 | 5.32E-16 |
| 4930500J02Rik | 0.982 | 8.008583 | 2.62E-17 | 1.45E-16 |
| Fsd1 | 0.982 | 8.74475 | 5.15E-17 | 2.01E-16 |
| Sox2ot | 0.982 | 6.9965 | 2.34E-14 | 2.81E-14 |
| Igsf21 | 0.978333 | 9.97175 | 6.24E-15 | 8.30E-15 |
| Gml | 0.976333 | 7.27425 | 2.53E-16 | 5.61E-16 |
| Gnat2 | 0.965 | 7.1285 | 1.05E-15 | 1.72E-15 |
| Cyct | 0.959333 | 12.46083 | 5.20E-15 | 7.05E-15 |
| Rffl | 0.954 | 8.387694 | 5.17E-17 | 2.01E-16 |
| Pmaip1 | 0.951333 | 9.219333 | 2.11E-16 | 5.08E-16 |
| Prex1 | 0.944667 | 10.15842 | 1.82E-17 | 1.32E-16 |
| Gldc | 0.938333 | 9.805333 | 4.92E-14 | 5.59E-14 |
| Tex21 | 0.92 | 7.648583 | 6.42E-14 | 7.08E-14 |
| Zbtb43 | 0.915167 | 10.13713 | 2.07E-16 | 5.05E-16 |
| 5730510P18Rik | 0.909 | 7.122583 | 4.06E-15 | 5.65E-15 |
| Cirh1a | 0.886556 | 8.902111 | 6.05E-17 | 2.16E-16 |
| Mup4 | 0.882 | 7.17625 | 2.23E-16 | 5.27E-16 |
| Ppan | 0.881667 | 13.09217 | 3.82E-19 | 1.61E-17 |
| Mns1 | 0.866333 | 11.88925 | 3.44E-17 | 1.58E-16 |
| Tcfl5 | 0.865 | 9.327667 | 3.89E-16 | 7.74E-16 |
| Ash2l | 0.855 | 11.96829 | 1.62E-19 | 1.09E-17 |
| Vwf | 0.854833 | 9.055167 | 1.72E-16 | 4.47E-16 |
| Arid3b | 0.853778 | 9.446 | 2.06E-17 | 1.33E-16 |
| Brdt | 0.848333 | 9.287208 | 7.88E-18 | 9.50E-17 |
| Ooep | 0.832 | 9.581417 | 1.05E-15 | 1.72E-15 |
| Gtsf1 | 0.827167 | 12.10458 | 2.86E-16 | 6.20E-16 |
| Ucma | 0.824333 | 7.23875 | 4.58E-15 | 6.33E-15 |
| Rnase6 | 0.819667 | 7.347833 | 8.92E-16 | 1.49E-15 |
| Rrad | 0.813 | 8.901083 | 6.51E-15 | 8.61E-15 |
| Treml4 | 0.805667 | 6.952583 | 4.96E-16 | 9.29E-16 |
| Katnal2 | 0.798667 | 8.785708 | 8.42E-17 | 2.75E-16 |
| Jph3 | 0.798 | 7.043833 | 1.18E-16 | 3.39E-16 |
| Henmt1 | 0.786667 | 7.393583 | 1.92E-16 | 4.84E-16 |
| 2410003L11Rik | 0.784 | 7.008 | 2.18E-16 | 5.19E-16 |
| Gm5622 | 0.779833 | 7.049625 | 5.33E-14 | 5.99E-14 |
| Fgf8 | 0.779333 | 8.034 | 1.18E-14 | 1.48E-14 |
| Dhx35 | 0.771333 | 11.34492 | 8.99E-19 | 2.77E-17 |
| Atg16l2 | 0.769333 | 8.4615 | 5.31E-14 | 5.99E-14 |
| Acad12 | 0.768 | 8.238042 | 2.75E-17 | 1.46E-16 |
| Gtf2a1l | 0.768 | 7.407833 | 1.45E-16 | 4.04E-16 |
| Wdr95 | 0.766333 | 7.3215 | 3.89E-16 | 7.74E-16 |
| Abcc8 | 0.742 | 7.3315 | 3.21E-15 | 4.61E-15 |
| 1700084M14Rik | 0.739333 | 6.98575 | 2.14E-16 | 5.14E-16 |
| Lhx5 | 0.739 | 6.689917 | 1.05E-14 | 1.33E-14 |
| Myo5c | 0.733333 | 6.9605 | 1.38E-15 | 2.19E-15 |
| Trpv4 | 0.732333 | 8.633 | 5.11E-16 | 9.39E-16 |
| Gdf3 | 0.731333 | 7.453167 | 3.25E-07 | 3.25E-07 |
| Wdr75 | 0.729 | 11.9775 | 1.97E-19 | 1.09E-17 |
| Siah2 | 0.728667 | 9.241917 | 3.29E-16 | 6.80E-16 |
| Dynlrb2 | 0.725 | 7.372667 | 4.83E-17 | 1.94E-16 |
| Reln | 0.72 | 8.441333 | 1.52E-14 | 1.90E-14 |
| Ctdsp2 | 0.718444 | 11.59347 | 1.17E-19 | 1.09E-17 |
| Mgl2 | 0.715 | 7.0255 | 3.17E-16 | 6.61E-16 |
| Dedd2 | 0.711417 | 9.310146 | 1.28E-17 | 1.21E-16 |
| 1700020N15Rik | 0.702667 | 7.750917 | 7.14E-17 | 2.43E-16 |
| Art5 | 0.699 | 8.060417 | 9.42E-17 | 2.98E-16 |
| Plb1 | 0.680333 | 7.05575 | 9.90E-17 | 3.01E-16 |
| Serpine1 | 0.677 | 7.4725 | 4.26E-17 | 1.83E-16 |
| Krt23 | 0.675 | 7.344083 | 2.24E-16 | 5.27E-16 |
| Ptgr1 | 0.672333 | 13.72883 | 8.32E-16 | 1.42E-15 |
| Dusp26 | 0.672 | 8.7875 | 3.45E-14 | 4.01E-14 |
| Dppa5a | 0.667833 | 12.54142 | 9.16E-15 | 1.17E-14 |
| Pnpla7 | 0.665667 | 10.45742 | 4.95E-18 | 7.65E-17 |
| Padi6 | 0.663333 | 7.441833 | 6.24E-16 | 1.11E-15 |
| Iqcg | 0.659667 | 11.44025 | 3.66E-15 | 5.18E-15 |
| Fbxo41 | 0.658333 | 7.269292 | 9.36E-16 | 1.55E-15 |
| Ssxb2 | 0.651 | 7.0165 | 4.72E-15 | 6.51E-15 |
| Mast1 | 0.649333 | 7.962917 | 1.13E-16 | 3.27E-16 |
| Tekt1 | 0.649167 | 8.47025 | 2.00E-16 | 5.00E-16 |
| Slc2a3 | 0.627 | 9.63325 | 1.29E-17 | 1.21E-16 |
| 3110002H16Rik | 0.621333 | 8.66325 | 7.83E-16 | 1.34E-15 |
| Rasal1 | 0.620667 | 7.369417 | 4.79E-16 | 9.07E-16 |
| Khdc3 | 0.616333 | 7.49575 | 6.54E-14 | 7.19E-14 |
| Plekhf2 | 0.611333 | 9.358417 | 3.43E-17 | 1.58E-16 |
| Kdm1b | 0.604333 | 12.22742 | 7.92E-17 | 2.66E-16 |
| 4933428M09Rik | 0.604333 | 6.659917 | 3.50E-16 | 7.19E-16 |
| 1700039O17Rik | 0.599 | 6.690667 | 3.10E-16 | 6.56E-16 |
| 3830417A13Rik | 0.596 | 6.95825 | 2.10E-16 | 5.08E-16 |
| Actn3 | 0.594667 | 8.449833 | 9.74E-17 | 3.01E-16 |
| Prkcq | 0.592 | 7.501125 | 1.59E-16 | 4.31E-16 |
| Ssxb1 | 0.589 | 6.857583 | 2.26E-15 | 3.41E-15 |
| Vil1 | 0.584 | 7.615167 | 2.37E-15 | 3.55E-15 |
| Kctd18 | 0.581667 | 8.242917 | 1.78E-16 | 4.55E-16 |
| Enho | 0.579 | 11.50667 | 3.30E-18 | 6.37E-17 |
| Zscan18 | 0.575333 | 9.125583 | 2.97E-18 | 6.11E-17 |
| Abcb6 | 0.574 | 10.67442 | 5.63E-14 | 6.30E-14 |
| Mlph | 0.569667 | 6.695583 | 1.58E-14 | 1.95E-14 |
| Capsl | 0.568667 | 7.715917 | 2.54E-15 | 3.72E-15 |
| Bzrap1 | 0.564 | 8.2765 | 3.14E-16 | 6.59E-16 |
| Arf3 | 0.563167 | 12.46554 | 2.28E-18 | 5.19E-17 |
| Mup3 | 0.562667 | 7.2375 | 1.73E-16 | 4.47E-16 |
| Was | 0.5615 | 7.492292 | 2.94E-15 | 4.25E-15 |
| Unc93a | 0.559333 | 7.125667 | 2.22E-14 | 2.69E-14 |
| Rhoh | 0.549333 | 7.203167 | 3.32E-17 | 1.58E-16 |
| Tfap2c | 0.536333 | 8.310417 | 1.74E-13 | 1.86E-13 |
| Mszf81 | 0.536 | 9.478833 | 5.08E-15 | 6.92E-15 |
| Slain1 | 0.534833 | 7.491417 | 5.00E-16 | 9.33E-16 |
| Dnmt3l | 0.534 | 8.09425 | 1.66E-15 | 2.55E-15 |
| Asphd2 | 0.529333 | 10.12733 | 1.42E-17 | 1.21E-16 |
| Gpsm3 | 0.523667 | 8.371583 | 2.64E-17 | 1.45E-16 |
| Ehd1 | 0.522667 | 13.76458 | 1.22E-19 | 1.09E-17 |
| Fggy | 0.510667 | 8.14275 | 1.76E-13 | 1.88E-13 |
| 4930558C23Rik | 0.509667 | 7.354667 | 3.29E-17 | 1.58E-16 |
| Ctag2 | 0.507 | 6.838417 | 1.68E-16 | 4.44E-16 |
| Rbm38 | 0.495 | 7.81 | 1.05E-16 | 3.14E-16 |
| Polr1e | 0.492 | 8.558167 | 2.33E-17 | 1.40E-16 |
| Rrp12 | 0.486333 | 8.186083 | 3.57E-16 | 7.29E-16 |
| Eya2 | 0.48 | 6.7795 | 2.83E-15 | 4.11E-15 |
| Tph1 | 0.478667 | 7.16675 | 4.01E-15 | 5.62E-15 |
| Ssxb3 | 0.471667 | 6.655333 | 6.21E-17 | 2.18E-16 |
| Ovol2 | 0.469333 | 6.939333 | 1.92E-17 | 1.32E-16 |
| Ccna1 | 0.463 | 6.819667 | 5.20E-17 | 2.01E-16 |
| Dcaf13 | 0.456667 | 10.95558 | 7.66E-18 | 9.50E-17 |
| BC026762 | 0.455 | 7.861833 | 5.65E-15 | 7.55E-15 |
| Car6 | 0.45 | 7.515 | 5.62E-15 | 7.53E-15 |
| Apba2 | 0.446667 | 7.249 | 1.30E-17 | 1.21E-16 |
| Prkab2 | 0.445333 | 9.589167 | 4.48E-17 | 1.88E-16 |
| 4930452B06Rik | 0.444778 | 8.56875 | 1.34E-17 | 1.21E-16 |
| Als2 | 0.438 | 7.664542 | 1.79E-17 | 1.32E-16 |
| Rsph1 | 0.438 | 7.862833 | 2.32E-17 | 1.40E-16 |
| Pacsin1 | 0.424889 | 6.950139 | 2.44E-16 | 5.55E-16 |
| Clgn | 0.424 | 9.0725 | 3.22E-14 | 3.79E-14 |
| Dpep3 | 0.419333 | 6.860833 | 3.11E-16 | 6.56E-16 |
| Cct8 | 0.416 | 13.67417 | 1.98E-19 | 1.09E-17 |
| Bcas1 | 0.413 | 6.964667 | 6.39E-16 | 1.13E-15 |
| Epb4.1l4a | 0.404 | 8.480417 | 4.68E-17 | 1.92E-16 |
| Prame | 0.403333 | 6.758 | 1.40E-15 | 2.20E-15 |
| 1700016K19Rik | 0.399333 | 7.783583 | 1.21E-16 | 3.44E-16 |
| Nlrp4f | 0.395 | 6.963833 | 2.61E-15 | 3.82E-15 |
| Ciapin1 | 0.392667 | 13.07583 | 4.28E-18 | 7.19E-17 |
| Mrpl23 | 0.388 | 14.59317 | 3.28E-20 | 1.09E-17 |
| Slc35f3 | 0.381667 | 7.873833 | 3.42E-17 | 1.58E-16 |
| 3110079O15Rik | 0.375 | 6.977333 | 2.39E-15 | 3.56E-15 |
| Ssx9 | 0.374333 | 6.737917 | 5.14E-16 | 9.39E-16 |
| Asz1 | 0.374333 | 10.16242 | 1.77E-16 | 4.55E-16 |
| Apoc2 | 0.369667 | 10.12983 | 2.34E-15 | 3.52E-15 |
| Muc3 | 0.362 | 7.012167 | 2.13E-14 | 2.59E-14 |
| Dnmt3a | 0.361667 | 10.26081 | 6.00E-18 | 8.13E-17 |
| Gm9895 | 0.359667 | 7.319417 | 4.00E-18 | 7.10E-17 |
| Piwil1 | 0.359333 | 6.6675 | 1.08E-17 | 1.15E-16 |
| Ccdc11 | 0.354333 | 7.253083 | 1.69E-16 | 4.44E-16 |
| Plekhg4 | 0.341667 | 11.19883 | 9.89E-17 | 3.01E-16 |
| Exd1 | 0.331667 | 10.28383 | 8.45E-15 | 1.09E-14 |
| Kcna1 | 0.327667 | 7.837667 | 4.35E-16 | 8.36E-16 |
| 1700086P04Rik | 0.320667 | 7.542333 | 1.80E-17 | 1.32E-16 |
| 1700012B07Rik | 0.32 | 6.995833 | 2.47E-16 | 5.55E-16 |
| Mtmr14 | 0.318167 | 9.247083 | 1.94E-18 | 4.70E-17 |
| Nfkbid | 0.316333 | 6.80425 | 2.80E-17 | 1.46E-16 |
| Otud7a | 0.312667 | 9.937667 | 2.93E-16 | 6.31E-16 |
| C130026I21Rik | 0.310333 | 6.721417 | 6.56E-16 | 1.15E-15 |
| Med24 | 0.31 | 11.60992 | 4.59E-19 | 1.61E-17 |
| Ppm1j | 0.308 | 7.776417 | 4.01E-16 | 7.90E-16 |
| Cd2 | 0.306 | 6.6575 | 1.66E-16 | 4.44E-16 |
| Rad51c | 0.305 | 8.457833 | 8.13E-16 | 1.39E-15 |
| 4933402E13Rik | 0.296333 | 7.498333 | 7.27E-16 | 1.26E-15 |
| Gulo | 0.296 | 7.883792 | 1.22E-17 | 1.21E-16 |
| Ldhc | 0.292333 | 9.48025 | 6.33E-15 | 8.39E-15 |
| Fabp9 | 0.292 | 6.911625 | 6.72E-16 | 1.17E-15 |
| Cnih3 | 0.289333 | 6.58925 | 5.68E-17 | 2.10E-16 |
| Mina | 0.287 | 12.58525 | 4.17E-17 | 1.83E-16 |
| Gsc | 0.286 | 6.799917 | 3.38E-15 | 4.83E-15 |
| Arrdc4 | 0.283 | 9.741125 | 2.66E-17 | 1.45E-16 |
| Slc34a3 | 0.279667 | 6.908 | 2.45E-17 | 1.43E-16 |
| Cdkn2b | 0.276667 | 8.095333 | 6.09E-16 | 1.09E-15 |
| 9530077C05Rik | 0.276 | 8.691083 | 4.79E-17 | 1.94E-16 |
| Prss45 | 0.267 | 6.697917 | 1.98E-17 | 1.32E-16 |
| Phf13 | 0.259667 | 12.64529 | 1.07E-16 | 3.14E-16 |
| Ntn1 | 0.257333 | 6.67425 | 9.13E-18 | 1.04E-16 |
| Cldn3 | 0.249667 | 7.143083 | 9.95E-18 | 1.10E-16 |
| Pla2g10 | 0.249 | 8.144333 | 1.70E-15 | 2.60E-15 |
| Nek5 | 0.238 | 6.804667 | 5.05E-16 | 9.37E-16 |
| Upb1 | 0.237667 | 7.7245 | 1.04E-14 | 1.32E-14 |
| Matn4 | 0.236333 | 6.7205 | 2.32E-16 | 5.36E-16 |
| D030056L22Rik | 0.227667 | 12.24108 | 6.11E-18 | 8.13E-17 |
| 1700001L19Rik | 0.227333 | 8.232 | 4.76E-15 | 6.53E-15 |
| Psmd2 | 0.221833 | 12.51454 | 9.54E-19 | 2.77E-17 |
| Kcnu1 | 0.220333 | 7.154667 | 2.83E-17 | 1.46E-16 |
| Chd1l | 0.219667 | 10.77558 | 4.52E-19 | 1.61E-17 |
| Ddx25 | 0.215 | 10.94588 | 3.79E-17 | 1.68E-16 |
| Esrp1 | 0.212333 | 7.1925 | 9.82E-16 | 1.62E-15 |
| Ctdp1 | 0.204333 | 8.667667 | 1.63E-14 | 2.00E-14 |
| Ahi1 | 0.1895 | 8.015042 | 1.95E-18 | 4.70E-17 |
| BC049635 | 0.188 | 6.903167 | 5.81E-16 | 1.05E-15 |
| Prss29 | 0.180667 | 6.670833 | 3.59E-16 | 7.30E-16 |
| Ctsc | 0.177667 | 9.583333 | 1.64E-17 | 1.32E-16 |
| Il12rb1 | 0.173333 | 7.397917 | 4.16E-16 | 8.09E-16 |
| Map3k5 | 0.170333 | 7.33625 | 1.44E-17 | 1.21E-16 |
| Syce1l | 0.165333 | 6.670667 | 3.84E-16 | 7.72E-16 |
| Nr2e1 | 0.164 | 6.501583 | 2.28E-16 | 5.32E-16 |
| 4930415O20Rik | 0.161667 | 6.870667 | 1.88E-17 | 1.32E-16 |
| Pkd2l1 | 0.158 | 6.794917 | 2.44E-17 | 1.43E-16 |
| Rab25 | 0.156333 | 6.649417 | 2.51E-17 | 1.44E-16 |
| Usp9y | 0.154667 | 6.588833 | 2.64E-16 | 5.81E-16 |
| Krt12 | 0.149333 | 6.610167 | 5.72E-17 | 2.10E-16 |
| Krt27 | 0.147 | 6.97275 | 3.19E-15 | 4.60E-15 |
| Nefm | 0.147 | 7.143667 | 2.12E-13 | 2.25E-13 |
| Fmn1 | 0.145667 | 7.510833 | 7.99E-17 | 2.66E-16 |
| 2310043M15Rik | 0.131333 | 6.701417 | 9.31E-16 | 1.55E-15 |
| Grb7 | 0.12 | 8.894 | 1.47E-15 | 2.29E-15 |
| Pcp4 | 0.116333 | 7.128167 | 1.31E-15 | 2.09E-15 |
| Tmem232 | 0.115 | 6.574333 | 1.81E-17 | 1.32E-16 |
| Myo16 | 0.114 | 6.497 | 2.87E-17 | 1.46E-16 |
| Cacng4 | 0.112667 | 7.603833 | 4.26E-17 | 1.83E-16 |
| Rhd | 0.112 | 10.34867 | 2.49E-13 | 2.64E-13 |
| Wdr17 | 0.108667 | 7.570833 | 4.09E-16 | 8.01E-16 |
| Trim61 | 0.108 | 6.770167 | 2.47E-16 | 5.55E-16 |
| Cyp4f39 | 0.104 | 6.5115 | 9.29E-17 | 2.96E-16 |
| Dgkg | 0.103 | 6.892 | 9.69E-17 | 3.01E-16 |
| Oas1d | 0.100333 | 6.911083 | 4.05E-18 | 7.10E-17 |
| Ceacam10 | 0.099667 | 6.7695 | 7.18E-17 | 2.43E-16 |
| Pik3r5 | 0.092 | 6.984583 | 5.16E-16 | 9.39E-16 |
| Mmel1 | 0.089333 | 8.129917 | 6.37E-14 | 7.04E-14 |
| Efcab6 | 0.088667 | 6.492583 | 2.95E-17 | 1.48E-16 |
| Clca3 | 0.088 | 6.917667 | 2.02E-16 | 5.01E-16 |
| C4bp-ps1 | 0.082333 | 6.684583 | 8.34E-18 | 9.76E-17 |
| Nphs1 | 0.077667 | 6.686 | 2.46E-16 | 5.55E-16 |
| 1700010D01Rik | 0.076 | 6.690583 | 6.17E-17 | 2.18E-16 |
| Ckmt1 | 0.073 | 7.125 | 5.56E-17 | 2.10E-16 |
| Ctsw | 0.069333 | 7.06925 | 4.54E-17 | 1.88E-16 |
| Kiss1 | 0.066333 | 6.762583 | 4.41E-17 | 1.87E-16 |
| Helt | 0.064667 | 6.519667 | 8.93E-16 | 1.49E-15 |
| Tmc5 | 0.064667 | 6.7875 | 5.81E-18 | 8.13E-17 |
| Cidec | 0.060333 | 6.621083 | 1.24E-16 | 3.49E-16 |
| Pnliprp2 | 0.059 | 6.887333 | 2.50E-16 | 5.57E-16 |
| Il1r2 | 0.057 | 6.761208 | 5.61E-17 | 2.10E-16 |
| Gzma | 0.054667 | 6.733583 | 3.59E-17 | 1.63E-16 |
| Slxl1 | 0.046 | 6.75425 | 1.99E-17 | 1.32E-16 |
| Igsf5 | 0.0455 | 6.8035 | 1.55E-17 | 1.28E-16 |
| Adipoq | 0.045333 | 6.647333 | 1.67E-17 | 1.32E-16 |
| Cdh15 | 0.033333 | 7.931667 | 1.30E-12 | 1.34E-12 |
| Mat1a | 0.031667 | 6.625 | 9.67E-17 | 3.01E-16 |
| Klc3 | 0.030667 | 9.852417 | 2.43E-16 | 5.55E-16 |
| Treml1 | 0.025333 | 8.01275 | 6.33E-16 | 1.13E-15 |
| Gsto2 | 0.0215 | 8.079792 | 1.53E-16 | 4.19E-16 |
| Cd300e | 0.02 | 6.670833 | 1.26E-17 | 1.21E-16 |
| Scg3 | 0.008 | 7.1725 | 6.61E-17 | 2.30E-16 |
| Rbpjl | 0.005333 | 6.6915 | 1.03E-16 | 3.12E-16 |
| Fgd2 | -0.001 | 8.014917 | 8.36E-17 | 2.75E-16 |
| A530040E14Rik | -0.01267 | 6.547667 | 2.64E-17 | 1.45E-16 |
| Gdpd3 | -0.01967 | 6.659833 | 5.11E-17 | 2.01E-16 |
| Cd86 | -0.02117 | 6.622167 | 6.32E-18 | 8.13E-17 |
| Serpinb5 | -0.03233 | 6.560333 | 7.18E-17 | 2.43E-16 |
| Arhgef16 | -0.03467 | 7.901333 | 4.84E-16 | 9.11E-16 |
| Arl13a | -0.035 | 6.664083 | 2.24E-17 | 1.39E-16 |
| Gm847 | -0.041 | 8.758417 | 1.11E-16 | 3.24E-16 |
| Gm10336 | -0.05233 | 10.48108 | 2.43E-19 | 1.17E-17 |
| Ube4b | -0.0765 | 8.701333 | 5.90E-17 | 2.15E-16 |
| Sly | -0.07833 | 6.88 | 8.46E-16 | 1.43E-15 |
| Zmynd10 | -0.082 | 6.897417 | 1.21E-16 | 3.44E-16 |
| Actn2 | -0.092 | 7.58075 | 6.95E-11 | 7.04E-11 |
| Tspan8 | -0.10567 | 7.71175 | 4.57E-16 | 8.74E-16 |
| Ripk4 | -0.10933 | 7.829917 | 5.15E-16 | 9.39E-16 |
| Ncan | -0.11167 | 7.77625 | 1.64E-15 | 2.53E-15 |
| 4933411K20Rik | -0.12533 | 10.72067 | 4.87E-18 | 7.65E-17 |
| Rhpn2 | -0.13433 | 9.746417 | 4.17E-16 | 8.09E-16 |
| Fgf21 | -0.16767 | 6.737083 | 1.42E-17 | 1.21E-16 |
| 4930422G04Rik | -0.20683 | 8.979792 | 8.56E-17 | 2.78E-16 |
| Dgka | -0.21167 | 10.52133 | 5.57E-18 | 8.13E-17 |
| Chl1 | -0.21267 | 7.207542 | 5.38E-17 | 2.06E-16 |
| Chd5 | -0.4145 | 7.753167 | 2.21E-17 | 1.39E-16 |
| Myom2 | -0.77533 | 8.505 | 1.77E-09 | 1.78E-09 |
| Baiap3 | -0.79667 | 7.412667 | 1.69E-16 | 4.44E-16 |
| Stmn4 | -1.03533 | 7.916 | 3.47E-13 | 3.65E-13 |
| Apobec2 | -1.104 | 8.327667 | 3.76E-10 | 3.80E-10 |
| Myh7 | -1.32517 | 10.41842 | 1.16E-09 | 1.17E-09 |
| Tnnc2 | -1.41667 | 8.590417 | 8.83E-08 | 8.85E-08 |
