# Supplementary material for: Sexual Fate Change of XX Germ Cells Caused by the Deletion of SMAD4 and STRA8 Independent of Somatic Sex Reprogramming
Source: PLoS Biol. 2016 Sep 8;14(9):e1002553. doi: 10.1371/journal.pbio.1002553 (PMC5015973; doi:10.1371/journal.pbio.1002553)
Supplement: S2 Table — (DOCX) [file pbio.1002553.s009.docx]

| ID | logFC | AveExpr | P.Value | adj.P.Val |
| --- | --- | --- | --- | --- |
| Inca1 | -4.00667 | 9.267667 | 1.62E-16 | 1.94E-16 |
| Dmrtc2 | -3.82133 | 10.61325 | 8.11E-14 | 8.30E-14 |
| Lypd4 | -3.762 | 8.754 | 6.39E-14 | 6.56E-14 |
| Ccdc79 | -3.73033 | 8.540333 | 1.76E-13 | 1.79E-13 |
| Reg2 | -3.571 | 8.185333 | 1.82E-11 | 1.83E-11 |
| Meiob | -3.439 | 10.8165 | 1.96E-17 | 2.79E-17 |
| Mei1 | -3.26333 | 10.23625 | 5.61E-13 | 5.66E-13 |
| Spdya | -3.11467 | 8.070083 | 2.85E-15 | 3.05E-15 |
| 1700028K03Rik | -3.04267 | 9.271333 | 3.42E-18 | 5.92E-18 |
| Syce3 | -2.97767 | 10.22825 | 7.61E-16 | 8.40E-16 |
| Dmc1 | -2.966 | 8.628833 | 4.98E-18 | 8.08E-18 |
| Zfp389 | -2.83433 | 9.38025 | 4.34E-17 | 5.66E-17 |
| Atf7ip2 | -2.77344 | 8.990444 | 4.04E-17 | 5.30E-17 |
| BC051142 | -2.72433 | 7.82625 | 1.11E-14 | 1.15E-14 |
| Pparg | -2.54267 | 8.5 | 7.76E-19 | 1.80E-18 |
| Tex12 | -2.535 | 12.06596 | 4.69E-18 | 7.68E-18 |
| 4933411G11Rik | -2.51933 | 7.770417 | 1.23E-15 | 1.34E-15 |
| Mtl5 | -2.40517 | 8.460583 | 6.88E-16 | 7.62E-16 |
| 1700123I01Rik | -2.38667 | 7.842417 | 4.78E-17 | 6.12E-17 |
| Acer3 | -2.23967 | 9.82275 | 6.27E-20 | 3.05E-19 |
| Fam72a | -2.23467 | 10.47267 | 1.31E-18 | 2.69E-18 |
| 4933406J08Rik | -2.23233 | 8.293333 | 3.48E-16 | 3.96E-16 |
| Tex101 | -2.216 | 14.16517 | 3.56E-17 | 4.71E-17 |
| H2-Q4 | -2.20867 | 10.66883 | 3.01E-19 | 8.87E-19 |
| 4930524B15Rik | -2.20233 | 7.59825 | 5.99E-18 | 9.33E-18 |
| Fgd6 | -2.193 | 11.71433 | 1.13E-18 | 2.42E-18 |
| Iqcb1 | -2.14867 | 11.82333 | 4.57E-20 | 2.56E-19 |
| Cyp2r1 | -2.13367 | 8.54075 | 4.53E-15 | 4.76E-15 |
| Ddb2 | -2.09133 | 9.72425 | 8.44E-20 | 3.66E-19 |
| 1700125H20Rik | -2.02667 | 8.57025 | 7.00E-15 | 7.35E-15 |
| Ccne2 | -2.02383 | 8.981958 | 1.02E-18 | 2.27E-18 |
| 4933427G17Rik | -2.01783 | 7.902417 | 1.75E-17 | 2.51E-17 |
| Xlr4b | -1.98733 | 11.85917 | 1.08E-17 | 1.58E-17 |
| Figla | -1.98067 | 9.809833 | 1.20E-13 | 1.22E-13 |
| Tppp | -1.975 | 7.610333 | 1.02E-16 | 1.25E-16 |
| 4932442L08Rik | -1.961 | 8.1055 | 3.93E-17 | 5.19E-17 |
| Hspb11 | -1.94167 | 12.771 | 5.75E-17 | 7.34E-17 |
| Stag3 | -1.915 | 12.36033 | 1.09E-17 | 1.59E-17 |
| H2-Q2 | -1.909 | 11.18958 | 3.59E-20 | 2.35E-19 |
| Syce1 | -1.888 | 12.4725 | 1.78E-19 | 6.22E-19 |
| Esco2 | -1.86367 | 10.96708 | 2.26E-19 | 7.40E-19 |
| Smc1b | -1.84 | 13.94058 | 3.17E-18 | 5.60E-18 |
| H2-Q7 | -1.837 | 10.39825 | 6.11E-19 | 1.47E-18 |
| Dip2c | -1.80633 | 9.695333 | 2.87E-20 | 2.03E-19 |
| Hormad1 | -1.79 | 10.01583 | 3.25E-19 | 9.50E-19 |
| 4930447C04Rik | -1.78592 | 10.41096 | 1.21E-18 | 2.52E-18 |
| Med21 | -1.77733 | 11.94433 | 1.41E-20 | 1.24E-19 |
| Asf1b | -1.77333 | 12.09067 | 1.35E-19 | 5.05E-19 |
| Xlr | -1.76967 | 11.06038 | 4.87E-18 | 7.93E-18 |
| Sdk1 | -1.76133 | 9.976417 | 7.22E-17 | 8.94E-17 |
| Ugt8a | -1.74878 | 7.533556 | 8.40E-19 | 1.94E-18 |
| Nmb | -1.747 | 11.06058 | 1.83E-18 | 3.57E-18 |
| Mlf1 | -1.74667 | 11.95375 | 7.88E-20 | 3.50E-19 |
| Ube2t | -1.738 | 13.407 | 2.14E-19 | 7.12E-19 |
| Fhl4 | -1.73383 | 8.155625 | 2.12E-17 | 2.97E-17 |
| BC049762 | -1.72567 | 8.027833 | 6.90E-17 | 8.60E-17 |
| Eaf2 | -1.71717 | 9.24625 | 5.97E-20 | 2.95E-19 |
| Cbwd1 | -1.71367 | 9.642042 | 3.38E-20 | 2.25E-19 |
| Haus8 | -1.71067 | 12.675 | 1.01E-19 | 4.12E-19 |
| Sycp3 | -1.70867 | 12.09267 | 3.41E-18 | 5.92E-18 |
| Rhox9 | -1.69833 | 11.7675 | 9.93E-15 | 1.04E-14 |
| Lpxn | -1.69767 | 8.273958 | 6.85E-17 | 8.57E-17 |
| Sycp2 | -1.69578 | 8.244194 | 3.41E-17 | 4.53E-17 |
| Adarb1 | -1.68878 | 8.958361 | 4.48E-19 | 1.20E-18 |
| Ankrd31 | -1.675 | 9.027667 | 2.49E-16 | 2.90E-16 |
| Spo11 | -1.67133 | 8.186333 | 4.37E-17 | 5.66E-17 |
| Tex16 | -1.65933 | 8.18475 | 6.26E-17 | 7.94E-17 |
| Cetn4 | -1.65833 | 9.497333 | 6.32E-17 | 7.98E-17 |
| Rab39 | -1.65 | 7.684333 | 1.62E-18 | 3.20E-18 |
| Lrrc34 | -1.64567 | 10.06417 | 1.02E-17 | 1.51E-17 |
| 0610042G04Rik | -1.632 | 8.595417 | 2.83E-16 | 3.27E-16 |
| Smpd3 | -1.62033 | 7.704528 | 5.87E-18 | 9.23E-18 |
| H2-T23 | -1.61633 | 10.43525 | 2.61E-19 | 8.25E-19 |
| Slc22a21 | -1.60767 | 8.726083 | 1.46E-19 | 5.37E-19 |
| Rbpms2 | -1.58667 | 12.54575 | 2.95E-18 | 5.28E-18 |
| 8030462N17Rik | -1.58067 | 7.458333 | 2.37E-17 | 3.25E-17 |
| Hist1h1a | -1.57467 | 8.697833 | 3.60E-16 | 4.09E-16 |
| Ccnb3 | -1.54567 | 8.068167 | 1.92E-14 | 1.99E-14 |
| Cdx2 | -1.53633 | 7.82375 | 8.74E-11 | 8.76E-11 |
| Ecsit | -1.533 | 13.15283 | 1.21E-21 | 3.70E-20 |
| Hormad2 | -1.52633 | 9.068 | 3.80E-18 | 6.43E-18 |
| Zcwpw1 | -1.5245 | 9.274875 | 2.46E-16 | 2.87E-16 |
| Phf7 | -1.518 | 9.476333 | 6.83E-18 | 1.06E-17 |
| Mettl4-ps1 | -1.51733 | 6.995083 | 2.20E-17 | 3.06E-17 |
| D630032N06Rik | -1.51333 | 7.896417 | 3.22E-18 | 5.65E-18 |
| Pigp | -1.51 | 13.74658 | 8.78E-19 | 2.01E-18 |
| Cyld | -1.50533 | 8.260167 | 2.48E-19 | 8.02E-19 |
| 1700001L05Rik | -1.5025 | 8.738042 | 3.98E-18 | 6.60E-18 |
| Amy1 | -1.49567 | 7.875167 | 2.65E-16 | 3.07E-16 |
| Il18 | -1.47967 | 8.561167 | 9.01E-16 | 9.89E-16 |
| Catsper2 | -1.479 | 9.18675 | 9.33E-20 | 3.90E-19 |
| H2-Ab1 | -1.47183 | 7.568146 | 3.53E-18 | 6.05E-18 |
| Spryd3 | -1.46333 | 11.04792 | 4.69E-20 | 2.56E-19 |
| Cdkl2 | -1.45833 | 8.19175 | 6.96E-17 | 8.64E-17 |
| Tgm1 | -1.445 | 7.320417 | 1.68E-16 | 2.00E-16 |
| H2-K1 | -1.43467 | 9.682444 | 2.56E-18 | 4.71E-18 |
| Adcy10 | -1.42667 | 7.831083 | 2.16E-14 | 2.23E-14 |
| 1700034F02Rik | -1.41267 | 7.320167 | 1.21E-16 | 1.46E-16 |
| Crb3 | -1.41067 | 8.280167 | 1.23E-15 | 1.34E-15 |
| Morn5 | -1.40733 | 8.303833 | 4.67E-16 | 5.26E-16 |
| Pcx | -1.39867 | 12.13667 | 4.81E-20 | 2.59E-19 |
| 1600016N20Rik | -1.39433 | 8.39225 | 1.59E-17 | 2.29E-17 |
| Sgsm3 | -1.39167 | 9.564333 | 5.15E-18 | 8.28E-18 |
| Sec11c | -1.381 | 9.168583 | 1.96E-18 | 3.74E-18 |
| Swt1 | -1.37844 | 9.902194 | 1.87E-18 | 3.62E-18 |
| Tuft1 | -1.372 | 10.695 | 1.51E-20 | 1.24E-19 |
| Rbmx2 | -1.35133 | 10.4375 | 5.38E-20 | 2.77E-19 |
| Dnajc21 | -1.34067 | 10.98633 | 1.15E-18 | 2.45E-18 |
| C330011M18Rik | -1.32867 | 9.1925 | 1.56E-14 | 1.63E-14 |
| Larp1b | -1.32333 | 10.14008 | 1.10E-19 | 4.41E-19 |
| 4933427D06Rik | -1.32267 | 7.765833 | 2.02E-17 | 2.86E-17 |
| Syngr4 | -1.32167 | 8.274333 | 9.41E-17 | 1.15E-16 |
| Atp11c | -1.32033 | 8.826417 | 1.46E-18 | 2.93E-18 |
| H2-D4 | -1.317 | 8.870917 | 9.59E-19 | 2.14E-18 |
| Stxbp5l | -1.317 | 7.086 | 2.10E-17 | 2.95E-17 |
| 3110057O12Rik | -1.31289 | 7.651278 | 2.97E-19 | 8.85E-19 |
| Syn2 | -1.31244 | 8.312583 | 5.37E-19 | 1.34E-18 |
| Prss16 | -1.309 | 7.354333 | 2.38E-17 | 3.25E-17 |
| Slc22a5 | -1.30517 | 8.7885 | 4.04E-20 | 2.38E-19 |
| Baz1a | -1.288 | 9.091167 | 1.85E-18 | 3.60E-18 |
| H2-Q10 | -1.2855 | 8.492 | 4.71E-19 | 1.23E-18 |
| Pdlim5 | -1.27693 | 7.65245 | 1.88E-19 | 6.48E-19 |
| Msh4 | -1.27433 | 7.746167 | 2.48E-18 | 4.62E-18 |
| 2310061I04Rik | -1.27333 | 11.61417 | 1.61E-19 | 5.67E-19 |
| Grk4 | -1.27133 | 7.781417 | 2.85E-16 | 3.27E-16 |
| Fmr1 | -1.259 | 11.31742 | 4.99E-20 | 2.64E-19 |
| Gpr160 | -1.256 | 8.244083 | 3.04E-18 | 5.39E-18 |
| Btbd10 | -1.2535 | 10.49404 | 1.50E-18 | 3.00E-18 |
| BC022960 | -1.25167 | 8.48775 | 2.33E-16 | 2.74E-16 |
| Fmr1nb | -1.245 | 10.70717 | 1.83E-17 | 2.61E-17 |
| Sgpl1 | -1.24183 | 10.80588 | 1.32E-21 | 3.70E-20 |
| Garnl3 | -1.241 | 9.486042 | 2.56E-19 | 8.15E-19 |
| Luzp1 | -1.23933 | 10.58125 | 3.88E-20 | 2.37E-19 |
| Hprt | -1.22867 | 14.175 | 1.42E-21 | 3.70E-20 |
| Msh5 | -1.22678 | 7.394944 | 4.54E-19 | 1.20E-18 |
| Ccdc155 | -1.21867 | 7.564583 | 8.90E-18 | 1.35E-17 |
| Pde6d | -1.21767 | 11.48867 | 1.15E-20 | 1.14E-19 |
| Acss2 | -1.21644 | 8.232778 | 1.91E-19 | 6.48E-19 |
| Papolg | -1.21567 | 10.96875 | 6.00E-19 | 1.45E-18 |
| Ubxn2a | -1.205 | 9.959167 | 1.56E-18 | 3.10E-18 |
| Chchd6 | -1.20467 | 13.70092 | 1.64E-21 | 3.70E-20 |
| Asb1 | -1.187 | 8.690167 | 1.51E-20 | 1.24E-19 |
| Ccdc41 | -1.18533 | 9.0375 | 9.61E-21 | 1.09E-19 |
| Nacad | -1.18333 | 7.4755 | 3.43E-19 | 9.84E-19 |
| Zfyve19 | -1.179 | 11.04083 | 4.40E-21 | 6.88E-20 |
| Pacrg | -1.17867 | 7.675167 | 2.87E-18 | 5.17E-18 |
| Ccdc92 | -1.17633 | 11.00983 | 7.77E-20 | 3.50E-19 |
| Ces5a | -1.171 | 7.010083 | 2.37E-17 | 3.25E-17 |
| Ankrd49 | -1.16067 | 10.31292 | 3.77E-20 | 2.37E-19 |
| Ccdc96 | -1.15867 | 7.333583 | 3.66E-19 | 1.03E-18 |
| Iqsec1 | -1.15467 | 9.265125 | 2.21E-19 | 7.30E-19 |
| Odf2l | -1.15467 | 10.7955 | 3.98E-18 | 6.60E-18 |
| Syce2 | -1.151 | 14.08892 | 1.21E-21 | 3.70E-20 |
| Zcwpw2 | -1.148 | 6.870083 | 1.21E-18 | 2.52E-18 |
| Gys2 | -1.14433 | 7.219417 | 4.19E-14 | 4.33E-14 |
| Sycp1 | -1.135 | 11.75625 | 3.80E-16 | 4.30E-16 |
| Slc25a25 | -1.12789 | 9.033583 | 1.08E-20 | 1.14E-19 |
| 4933416C03Rik | -1.12767 | 7.240167 | 3.78E-19 | 1.03E-18 |
| BC048679 | -1.12 | 9.260333 | 8.68E-17 | 1.06E-16 |
| Taf7l | -1.11917 | 11.43608 | 6.03E-16 | 6.74E-16 |
| Hfm1 | -1.108 | 7.554917 | 1.05E-17 | 1.54E-17 |
| Tc2n | -1.10617 | 6.95825 | 2.71E-19 | 8.47E-19 |
| Rhox6 | -1.106 | 11.07925 | 4.39E-17 | 5.67E-17 |
| Rbm27 | -1.10517 | 10.43946 | 2.29E-20 | 1.79E-19 |
| Zfp672 | -1.104 | 10.20658 | 2.61E-18 | 4.74E-18 |
| Lyrm1 | -1.104 | 8.353083 | 2.88E-19 | 8.72E-19 |
| Zkscan2 | -1.092 | 8.100875 | 3.00E-18 | 5.35E-18 |
| Spata5 | -1.08433 | 14.69625 | 2.34E-21 | 4.51E-20 |
| Ppp3cc | -1.083 | 9.310583 | 1.26E-19 | 4.88E-19 |
| Rnls | -1.08167 | 8.67575 | 5.62E-18 | 8.91E-18 |
| Pik3r3 | -1.08133 | 9.43175 | 2.78E-19 | 8.62E-19 |
| 4930432K21Rik | -1.07267 | 8.669708 | 1.96E-18 | 3.74E-18 |
| 6430548M08Rik | -1.06833 | 8.510972 | 4.37E-17 | 5.66E-17 |
| Ccdc122 | -1.06567 | 7.460167 | 2.17E-17 | 3.02E-17 |
| Tsc22d3 | -1.04722 | 10.92953 | 7.23E-20 | 3.34E-19 |
| Boll | -1.04167 | 7.63375 | 2.56E-18 | 4.71E-18 |
| Zdhhc13 | -1.03833 | 10.14754 | 4.48E-21 | 6.88E-20 |
| Nfxl1 | -1.03767 | 9.838583 | 1.53E-21 | 3.70E-20 |
| Ndufa1 | -1.03467 | 14.41142 | 5.68E-22 | 3.43E-20 |
| Ankrd34b | -1.02633 | 8.552167 | 3.09E-15 | 3.29E-15 |
| 4930481B07Rik | -1.01667 | 7.2865 | 9.54E-18 | 1.43E-17 |
| Zc3h8 | -1.015 | 9.0255 | 1.58E-16 | 1.89E-16 |
| Parp12 | -1.00967 | 8.632083 | 1.49E-19 | 5.37E-19 |
| Mkl2 | -1.00711 | 7.192833 | 1.21E-19 | 4.80E-19 |
| Phf16 | -0.99178 | 8.632694 | 1.17E-20 | 1.14E-19 |
| Hells | -0.99033 | 12.40508 | 9.43E-22 | 3.70E-20 |
| Kdm4d | -0.99033 | 8.427417 | 1.12E-17 | 1.63E-17 |
| Nrip3 | -0.987 | 7.389833 | 1.12E-18 | 2.42E-18 |
| Uba6 | -0.97975 | 8.253917 | 5.64E-20 | 2.86E-19 |
| Trappc2 | -0.97067 | 11.28867 | 2.70E-20 | 1.99E-19 |
| Fnip1 | -0.95933 | 9.801354 | 2.50E-20 | 1.88E-19 |
| Gabrr1 | -0.95667 | 7.676333 | 3.90E-16 | 4.41E-16 |
| 4930579C12Rik | -0.956 | 6.934417 | 1.05E-16 | 1.28E-16 |
| Tiparp | -0.95333 | 11.9175 | 4.59E-21 | 6.88E-20 |
| Stx16 | -0.94567 | 8.511583 | 2.81E-18 | 5.08E-18 |
| Tktl1 | -0.94233 | 9.214 | 9.22E-18 | 1.39E-17 |
| Etnk1 | -0.94222 | 7.989972 | 1.97E-18 | 3.74E-18 |
| Stra8 | -0.94 | 13.5915 | 5.39E-18 | 8.58E-18 |
| Trmt10a | -0.93317 | 10.28717 | 4.38E-20 | 2.54E-19 |
| S100pbp | -0.92956 | 9.573472 | 6.89E-20 | 3.27E-19 |
| Gpr19 | -0.92567 | 7.599667 | 1.30E-18 | 2.69E-18 |
| Dgat1 | -0.925 | 9.438875 | 5.40E-19 | 1.34E-18 |
| Hccs | -0.91133 | 11.64283 | 1.62E-21 | 3.70E-20 |
| Vma21 | -0.91 | 10.7325 | 4.33E-18 | 7.13E-18 |
| Glce | -0.90389 | 9.224083 | 2.17E-21 | 4.51E-20 |
| Avl9 | -0.898 | 10.04033 | 2.38E-21 | 4.51E-20 |
| Sirt1 | -0.89767 | 10.98008 | 3.20E-22 | 3.43E-20 |
| Rnf141 | -0.89067 | 9.081146 | 1.25E-20 | 1.16E-19 |
| Il4i1 | -0.88833 | 7.875083 | 3.20E-16 | 3.65E-16 |
| Hdgfrp2 | -0.88633 | 13.06083 | 7.43E-21 | 9.55E-20 |
| Ccdc158 | -0.88583 | 7.411708 | 5.26E-19 | 1.32E-18 |
| Angptl6 | -0.88383 | 9.821042 | 9.43E-19 | 2.13E-18 |
| Maoa | -0.88367 | 13.11558 | 5.46E-21 | 7.57E-20 |
| 1700013H16Rik | -0.877 | 11.23133 | 4.61E-17 | 5.92E-17 |
| Lrrc42 | -0.87383 | 11.77746 | 2.01E-20 | 1.61E-19 |
| Cspp1 | -0.87167 | 7.607167 | 2.16E-18 | 4.05E-18 |
| Sycp1-ps1 | -0.87133 | 9.510667 | 5.00E-14 | 5.14E-14 |
| Zcrb1 | -0.871 | 13.37392 | 2.51E-22 | 3.43E-20 |
| Calr3 | -0.865 | 7.886167 | 7.38E-19 | 1.73E-18 |
| Trappc8 | -0.85467 | 7.909583 | 1.91E-19 | 6.48E-19 |
| Zfp654 | -0.85433 | 9.166375 | 1.49E-19 | 5.37E-19 |
| 1700037C18Rik | -0.84633 | 8.381917 | 3.73E-19 | 1.03E-18 |
| Aspa | -0.84583 | 7.314458 | 2.40E-17 | 3.27E-17 |
| Slc25a42 | -0.83933 | 6.984667 | 1.04E-17 | 1.54E-17 |
| Gm15246 | -0.82867 | 7.54225 | 1.41E-16 | 1.69E-16 |
| Pfdn4 | -0.82667 | 8.534 | 5.26E-20 | 2.74E-19 |
| 5430410E06Rik | -0.81933 | 7.013167 | 8.61E-18 | 1.31E-17 |
| Gm13034 | -0.81833 | 11.4205 | 3.33E-18 | 5.81E-18 |
| Wdr44 | -0.81667 | 10.79992 | 5.69E-21 | 7.59E-20 |
| Nek1 | -0.81267 | 9.10175 | 1.03E-20 | 1.13E-19 |
| Macrod2 | -0.81233 | 7.713833 | 9.88E-18 | 1.47E-17 |
| Sdr42e1 | -0.812 | 10.466 | 5.18E-19 | 1.32E-18 |
| Htt | -0.80083 | 8.694667 | 6.39E-19 | 1.52E-18 |
| Fam92b | -0.79967 | 7.919083 | 3.22E-15 | 3.42E-15 |
| Ccdc82 | -0.7845 | 8.020625 | 8.70E-20 | 3.68E-19 |
| Esf1 | -0.78233 | 11.99444 | 9.83E-20 | 4.07E-19 |
| Tmem116 | -0.77983 | 7.137208 | 1.42E-16 | 1.71E-16 |
| Zmym6 | -0.774 | 10.17442 | 7.06E-20 | 3.30E-19 |
| Irx2 | -0.76783 | 7.32825 | 7.94E-18 | 1.22E-17 |
| Tmem129 | -0.765 | 8.824583 | 4.62E-20 | 2.56E-19 |
| Tdrkh | -0.75933 | 9.528917 | 9.52E-21 | 1.09E-19 |
| Efcab2 | -0.75633 | 9.49225 | 3.66E-18 | 6.21E-18 |
| Cstf2 | -0.75633 | 9.005833 | 4.45E-19 | 1.20E-18 |
| Ccdc150 | -0.7485 | 7.260125 | 3.46E-18 | 5.95E-18 |
| Pls1 | -0.74667 | 8.890667 | 3.40E-17 | 4.53E-17 |
| Rapgef5 | -0.737 | 8.580896 | 5.95E-18 | 9.31E-18 |
| Hus1b | -0.73467 | 7.870083 | 1.98E-18 | 3.74E-18 |
| Xrcc4 | -0.73133 | 9.405833 | 2.81E-20 | 2.02E-19 |
| Ccdc101 | -0.71733 | 10.81475 | 1.58E-19 | 5.62E-19 |
| Eif1ad | -0.71317 | 10.80129 | 3.09E-20 | 2.14E-19 |
| Brca2 | -0.71283 | 9.860042 | 2.92E-19 | 8.76E-19 |
| Prss50 | -0.71083 | 7.039 | 5.23E-18 | 8.36E-18 |
| Pabpc6 | -0.707 | 6.944583 | 1.06E-18 | 2.32E-18 |
| Wdr31 | -0.70133 | 9.6575 | 2.50E-18 | 4.63E-18 |
| Camta1 | -0.694 | 11.74892 | 5.47E-21 | 7.57E-20 |
| Cox5a | -0.68567 | 15.52017 | 1.47E-20 | 1.24E-19 |
| Dhrs2 | -0.68533 | 7.11875 | 1.42E-15 | 1.54E-15 |
| Rps6ka6 | -0.68033 | 8.515917 | 1.27E-19 | 4.88E-19 |
| 1700029J07Rik | -0.678 | 8.8225 | 5.84E-20 | 2.92E-19 |
| Pde3b | -0.673 | 7.225333 | 1.21E-18 | 2.52E-18 |
| Syt14 | -0.67283 | 6.951542 | 2.81E-19 | 8.65E-19 |
| H2-Bl | -0.67011 | 7.306417 | 5.79E-18 | 9.15E-18 |
| Pex7 | -0.65733 | 11.33625 | 2.48E-20 | 1.88E-19 |
| Terf1 | -0.65133 | 11.6525 | 3.96E-20 | 2.37E-19 |
| Rnf17 | -0.65133 | 8.022583 | 3.26E-17 | 4.38E-17 |
| Spata1 | -0.65033 | 8.209417 | 1.13E-18 | 2.42E-18 |
| Lrrc46 | -0.64917 | 7.373875 | 6.85E-17 | 8.57E-17 |
| Ube2a | -0.64567 | 11.53 | 5.72E-22 | 3.43E-20 |
| Gbp3 | -0.63867 | 8.3885 | 9.76E-16 | 1.06E-15 |
| 2310010J17Rik | -0.63567 | 12.61375 | 3.75E-20 | 2.37E-19 |
| Dmrtc1c1 | -0.62933 | 9.61875 | 1.81E-16 | 2.14E-16 |
| Dmrta2 | -0.62167 | 7.31025 | 2.67E-17 | 3.61E-17 |
| Txlng | -0.6185 | 9.048083 | 3.93E-20 | 2.37E-19 |
| Ybx2 | -0.618 | 8.7765 | 6.35E-16 | 7.08E-16 |
| H2bfm | -0.614 | 7.171583 | 2.16E-18 | 4.05E-18 |
| Lrriq3 | -0.61133 | 7.019125 | 8.44E-20 | 3.66E-19 |
| Tinf2 | -0.597 | 8.618583 | 1.24E-19 | 4.85E-19 |
| Gm711 | -0.59633 | 6.794167 | 3.78E-19 | 1.03E-18 |
| Kidins220 | -0.593 | 7.296333 | 8.84E-18 | 1.34E-17 |
| D1Pas1 | -0.59183 | 8.525292 | 6.45E-20 | 3.10E-19 |
| 2610100L16Rik | -0.59033 | 6.874292 | 1.20E-18 | 2.52E-18 |
| 1700031C06Rik | -0.58367 | 7.985333 | 5.21E-19 | 1.32E-18 |
| Phf8 | -0.5725 | 9.7995 | 9.65E-21 | 1.09E-19 |
| Ly6k | -0.57233 | 7.520167 | 8.61E-18 | 1.31E-17 |
| Celf6 | -0.55833 | 7.293083 | 1.06E-18 | 2.32E-18 |
| Myt1l | -0.55467 | 7.352667 | 5.99E-19 | 1.45E-18 |
| Nol8 | -0.552 | 9.900375 | 8.65E-17 | 1.06E-16 |
| Nme5 | -0.54967 | 7.6325 | 4.70E-19 | 1.23E-18 |
| Unc13a | -0.54917 | 7.300958 | 6.40E-18 | 9.94E-18 |
| Elavl3 | -0.549 | 7.148167 | 1.36E-18 | 2.76E-18 |
| Stard6 | -0.54867 | 7.297 | 3.95E-19 | 1.07E-18 |
| 4930573O21Rik | -0.54267 | 7.1185 | 6.75E-17 | 8.49E-17 |
| Nr6a1 | -0.541 | 8.839417 | 4.00E-18 | 6.61E-18 |
| Utp18 | -0.52967 | 9.248417 | 1.25E-20 | 1.16E-19 |
| Ofd1 | -0.529 | 13.69917 | 3.20E-20 | 2.17E-19 |
| Efcab5 | -0.519 | 6.74825 | 3.90E-18 | 6.54E-18 |
| Abcb11 | -0.50933 | 6.935083 | 1.18E-10 | 1.18E-10 |
| Gkap1 | -0.50667 | 11.94921 | 2.93E-21 | 5.03E-20 |
| Gls2 | -0.50367 | 8.874833 | 7.39E-19 | 1.73E-18 |
| Yrdc | -0.47917 | 9.759458 | 8.04E-21 | 9.99E-20 |
| 4930564K09Rik | -0.46933 | 6.869333 | 5.03E-19 | 1.30E-18 |
| Lrrc6 | -0.46833 | 6.926417 | 7.53E-20 | 3.43E-19 |
| Mysm1 | -0.4605 | 8.401542 | 1.17E-20 | 1.14E-19 |
| Hsf2bp | -0.456 | 6.867833 | 5.13E-18 | 8.28E-18 |
| Nxnl1 | -0.44367 | 6.606333 | 6.45E-19 | 1.53E-18 |
| Poln | -0.43433 | 6.82275 | 1.29E-19 | 4.89E-19 |
| 1700028J19Rik | -0.43267 | 6.78575 | 2.60E-18 | 4.74E-18 |
| Hist1h3a | -0.43133 | 8.004 | 2.06E-17 | 2.91E-17 |
| Ocel1 | -0.42267 | 10.33917 | 4.38E-15 | 4.62E-15 |
| Zfp622 | -0.421 | 11.6795 | 3.73E-19 | 1.03E-18 |
| Siah1b | -0.41717 | 12.32813 | 3.92E-22 | 3.43E-20 |
| Uchl3 | -0.40933 | 12.62783 | 8.01E-22 | 3.70E-20 |
| 4933430I17Rik | -0.39933 | 7.889917 | 8.69E-20 | 3.68E-19 |
| Pmel | -0.39667 | 7.21175 | 5.61E-19 | 1.37E-18 |
| Cdkn2a | -0.38367 | 7.514083 | 1.52E-15 | 1.64E-15 |
| Aoah | -0.38133 | 6.774583 | 1.48E-19 | 5.37E-19 |
| Emd | -0.373 | 13.17483 | 2.70E-21 | 4.86E-20 |
| Opn3 | -0.367 | 8.75325 | 4.17E-17 | 5.46E-17 |
| H2-Q1 | -0.3645 | 9.111708 | 5.19E-19 | 1.32E-18 |
| Tex13 | -0.35933 | 10.11925 | 7.81E-17 | 9.63E-17 |
| 4930558K02Rik | -0.34 | 6.667917 | 1.82E-18 | 3.57E-18 |
| Morn2 | -0.33867 | 8.866583 | 1.02E-19 | 4.14E-19 |
| Spaca1 | -0.33583 | 8.887833 | 6.64E-16 | 7.38E-16 |
| Slc5a10 | -0.33167 | 8.718083 | 9.52E-19 | 2.14E-18 |
| Abcc2 | -0.331 | 6.824167 | 1.75E-17 | 2.51E-17 |
| BC061194 | -0.31067 | 6.717417 | 9.04E-19 | 2.06E-18 |
| Abcb7 | -0.30667 | 8.867667 | 1.49E-20 | 1.24E-19 |
| Styxl1 | -0.30133 | 6.800667 | 1.37E-18 | 2.76E-18 |
| 8030474K03Rik | -0.29467 | 9.816 | 2.51E-17 | 3.40E-17 |
| Lrrc51 | -0.28533 | 7.983375 | 5.15E-16 | 5.78E-16 |
| Tsks | -0.28467 | 6.872167 | 2.86E-19 | 8.72E-19 |
| Gbx2 | -0.272 | 7.149083 | 9.27E-16 | 1.01E-15 |
| Rhox11 | -0.27133 | 6.619583 | 3.45E-19 | 9.84E-19 |
| Gpr137c | -0.27033 | 7.013417 | 1.32E-18 | 2.69E-18 |
| Suv39h1 | -0.26433 | 10.31175 | 2.50E-19 | 8.02E-19 |
| 1700018B24Rik | -0.26133 | 9.691125 | 2.80E-17 | 3.78E-17 |
| Gm16432 | -0.254 | 6.975667 | 3.92E-13 | 3.97E-13 |
| Dnajc5g | -0.231 | 7.181542 | 2.36E-17 | 3.25E-17 |
| Nptx2 | -0.22567 | 7.321 | 6.18E-17 | 7.86E-17 |
| Ribc1 | -0.218 | 9.649667 | 3.43E-19 | 9.84E-19 |
| Adam22 | -0.20267 | 6.5675 | 1.98E-19 | 6.67E-19 |
| Wbp2nl | -0.19033 | 8.546583 | 1.79E-16 | 2.12E-16 |
| Alg13 | -0.17189 | 9.971861 | 4.47E-20 | 2.56E-19 |
| Myh4 | -0.14117 | 7.643792 | 1.06E-18 | 2.32E-18 |
| 1700065D16Rik | -0.138 | 6.61525 | 3.84E-18 | 6.46E-18 |
| Mtg1 | -0.13767 | 11.27092 | 1.26E-21 | 3.70E-20 |
| 1700026N04Rik | -0.08333 | 6.573333 | 3.48E-19 | 9.85E-19 |
| Gm4779 | -0.05333 | 10.193 | 9.90E-18 | 1.47E-17 |
| Fra10ac1 | -0.01133 | 13.397 | 4.78E-22 | 3.43E-20 |
| Pla2g2c | 0.138333 | 8.70175 | 1.02E-13 | 1.04E-13 |
| AI854517 | 0.211667 | 8.439917 | 3.59E-18 | 6.12E-18 |
| Rhox2a | 0.217333 | 11.43967 | 3.30E-15 | 3.49E-15 |
| Nkx2-4 | 0.2225 | 7.77175 | 5.56E-19 | 1.37E-18 |
| Gm7361 | 0.244667 | 8.151667 | 2.85E-16 | 3.27E-16 |
| Slc27a2 | 0.307333 | 8.1295 | 2.39E-16 | 2.80E-16 |
| Tcte3 | 0.318333 | 8.075625 | 2.53E-16 | 2.94E-16 |
| Sohlh2 | 0.372667 | 9.5225 | 1.55E-15 | 1.66E-15 |
| Lhx8 | 0.485 | 7.323042 | 2.14E-15 | 2.29E-15 |
| Hsf5 | 0.655333 | 9.59375 | 8.28E-16 | 9.11E-16 |
| Rhox3a | 0.918667 | 7.502417 | 3.37E-17 | 4.51E-17 |
