# Supplementary material for: Sexual Fate Change of XX Germ Cells Caused by the Deletion of SMAD4 and STRA8 Independent of Somatic Sex Reprogramming
Source: PLoS Biol. 2016 Sep 8;14(9):e1002553. doi: 10.1371/journal.pbio.1002553 (PMC5015973; doi:10.1371/journal.pbio.1002553)
Supplement: S3 Table — (DOCX) [file pbio.1002553.s010.docx]

|  | Forward (5'–3') | Reverse (5'–3') |
| --- | --- | --- |
| *Nanos2* | ACAGCAGTCAGCAGTCTC | CCGAGAAGTCATCACCAG |
| *Nodal* | AGCCAAGAAGAGGATCTGGTATGG | GACCTGAGAAGGAATGACGGTGAA |
| *Lefty1* | AGTCCTGGACAAGGCTGATGTG | CGAACACTAGCAGGTGAGTGGA |
| *Mvh* | GTTGAAGTATCTGGACATGATGCAC | CGAGTTGGTGCTACAATAATACACTC |
| *G3pdh* | ACCACAGTCCATGCCATCAC | TCCACCACCCTGTTGCTGTA |
| *Stra8* | CCTAAGGAAGGCAGTTTACTCCCAGTC | GCAGGTTGAAGGATGCTTTGAGC |
| *Sox9* | AAGACCACCCCGATTACAAGTACCA | TCAGATCAACTTTGCCAGCTTGCAC |
| *Uft1* | ATGTCCCGGTGACTACGTCTGATG | AGTCTCGGAGTTTGTCCTTGAGGAA |
| *Figla* | GCCCCTCCTCTTCTTTCTTCA | CAGAGCAGGAAGCCCAGTAAA |
| *Sox2* | GCGGAGTGGAAACTTTTGTCC | CGGGAAGCGTGTACTTATCCTT |
| *Nanog* | CCGCTTGCACTTCATCCTTTG | CCTCAGCCTCCAGCAGATGC |
| *Oct4 (pouf1)* | TCACCTTGGGGTACACCCAG | CATGTTCTTAAGGCTGAGCTGC |
| *Lhx8* | GTCAGTCCCAACCATTCTTCC | CTGGGCAGCGAGAGGATGTT |
| *Sohlh1* | AGCCAGACTCCGGTATAGCCA | CAAGCTGGAAGACTCTGGCT |
| *Smad4* | CCTGTTGTGACTGTGGATGGCTATG | AGACCTTTATATACGCGCTTGGGTAGA |
| *Rec8* | AGGAGACCCTTCCTGAAGCC | ATGGGCTCTGCCTCCTGTAA |
| *Topaz1* | ACATTGCGTGGCTGTGAGC | CTCAGGCACATGGACAAACTTG |
| *Piwil2* | TGACCTGTGCATCCCCTTCT | TCCCCACAAGCTTCATATCCA |
| *Egr4* | AGATCTGAGTTCGGCCGCCTTT | GACCTTGGTCCCTACTGCAGAGA |
| *Tdrd5* | AGGGGTCATATTCTACAGGATTCCC | CGCTTTAGCTCAGGATCAACAGTC |
| *Dnmt3l* | GACTACCAGAATGCTATGCGG | GCAATCAAACAGGAGAGAAAC |
| *Dnd1* | GTTCAGTACGCACCGAGCTG | GCGGAAGTGCTGCTTTAGGT |
| *Piwil4* | CGGGACGGTGTAGGCAATGG | GTCAGCTTGTGTGCATATTGGC |
| *Tdrd5* | ACAAGAGTCAGGGAGCCATAG | CACATCAAAGTAGTCAGTTAGCA |
